# Photophysical Study on the Rigid Pt(II) Complex [Pt(naphen)(Cl)] (Hnaphen = Naphtho[1,2-*b*][1,10]Phenanthroline and Derivatives

**DOI:** 10.3390/molecules27207022

**Published:** 2022-10-18

**Authors:** Maren Krause, Iván Maisuls, Stefan Buss, Cristian A. Strassert, Andreas Winter, Ulrich S. Schubert, Shruthi S. Nair, Benjamin Dietzek-Ivanšić, Axel Klein

**Affiliations:** 1 University of Cologne, Faculty of Mathematics and Natural Sciences, Department of Chemistry, Institute for Inorganic Chemistry, Greinstrasse 6, 50939 Köln, Germany; 2 Westfälische Wilhelms-Universität Münster, Institut für Anorganische und Analytische Chemie, CeNTech, CiMIC, SoN, Heisenbergstr. 11, 48149 Münster, Germany; 3 Laboratory of Organic and Macromolecular Chemistry (IOMC), Friedrich Schiller University Jena, Humboldtstr. 10, 07743 Jena, Germany; 4 Center for Energy and Environmental Chemistry Jena (CEEC Jena), Friedrich Schiller University Jena, Philosophenweg 7a, 07743 Jena, Germany; 5 Institute for Physical Chemistry (IPC), Friedrich Schiller University Jena, Helmholtzweg 4, 07743 Jena, Germany; 6 Leibniz Institute for Photonic Technologies Jena (IPHT), Albert-Einstein-Str. 9, 07745 Jena, Germany

**Keywords:** platinum, cyclometalation, pericyclic ligands, photoluminescence, time-resolved

## Abstract

The electrochemistry and photophysics of the Pt(II) complexes [Pt(naphen)(X)] (Hnaphen = naphtho[1,2-*b*][1,10]phenanthroline, X = Cl or C≡CPh) containing the rigid tridentate *C^N^N*-coordinating pericyclic naphen ligand was studied alongside the complexes of the tetrahydro-derivative [Pt(thnaphen)(X)] (Hthnaphen = 5,6,8,9-tetrahydro-naphtho[1,2-*b*][1,10]phenanthroline) and the *N^C^N*-coordinated complex [Pt(bdq)(Cl)] (Hbdq = benzo[1,2-*h*:5,4-*h*’]diquinoline. The cyclic voltammetry showed reversible reductions for the *C^N^N* complexes, with markedly fewer negative potentials (around −1.6 V vs. ferrocene) for the complexes containing the naphen ligand compared with the thnaphen derivatives (around −1.9 V). With irreversible oxidations at around +0.3 V for all of the complexes, the naphen made a difference in the electrochemical gap of about 0.3 eV (1.9 vs. 2.2 eV) compared with thnaphen. The bdq complex was completely different, with an irreversible reduction at around −2 V caused by the *N^C^N* coordination pattern, which lacked a good electron acceptor such as the phenanthroline unit in the *C^N^N* ligand naphen. Long-wavelength UV-Vis absorption bands were found around 520 to 530 nm for the *C^N^N* complexes with the C≡CPh coligand and were red-shifted when compared with the Cl derivatives. The *N^C^N*-coordinated bdq complex was markedly blue-shifted (493 nm). The steady-state photoluminescence spectra showed poorly structured emission bands peaking at around 630 nm for the two naphen complexes and 570 nm for the thnaphen derivatives. The bdq complex showed a pronounced vibrational structure and an emission maximum at 586 nm. Assuming mixed ^3^LC/^3^MLCT excited states, the vibronic progression for the *N^C^N* bdq complex indicated a higher LC character than assumed for the *C^N^N*-coordinated naphen and thnaphen complexes. The blue-shift was a result of the different *N^C^N* vs. *C^N^N* coordination. The photoluminescence lifetimes and quantum yields *Φ*_L_ massively increased from solutions at 298 K (0.06 to 0.24) to glassy frozen matrices at 77 K (0.80 to 0.95). The nanosecond time-resolved study on [Pt(naphen)(Cl)] showed a phosphorescence emission signal originating from the mixed ^3^LC/^3^MLCT with an emission lifetime of around 3 µs.

## 1. Introduction

The study of luminescent materials based on transition metal complexes is motivated by a number of importantapplications in fields such as photocatalysis [1,2,3], sensing and bioimaging [4,5,6,7], and optoelectronic devices [8,9,10,11,12,13,14,15,16]. In the field of OLEDs (organic light emitting diodes), phosphorescent metal complexes are of particular interest due to their ability to harvest all generated excitons through the availability of excited triplet states. The necessary intersystem crossing (ISC) is facilitated through efficient spin-orbit coupling (SOC) associated with the heavy metal centres, which also boosts the phosphorescent relaxation [8,10,17]. Typical phosphorescent (triplet) emitters are based on metal cations such as Ir(III), Ru(II), and Pt(II) [4,5,6,7,8,9,10,11,12,13,14,15,16]. While the d^6^ metal centres of Ir(III) and Ru(II) adopt an octahedral coordination environment, the d^8^ configured Pt(II) complexes and the less frequently used Pd(II) and Au(III) coordination compounds adopt square planar geometries with open coordination sites in the axial positions [16,18]. These open axial flanks can lead to metal–metal (M···M) and/or π−π stacking interactions in the aggregates with red-shifted emission originating from MMLCT (metal–metal–ligand charge transfer) states or the LC (ligand-centred) character of monomers [8,10,14,18,19,20,21,22,23,24,25].

For the design of efficient phosphorescent Pt(II) complexes, two main structural features have turned out to be beneficial. First, a rigid coordination sphere prevents radiationless decay from the triplet excited states, while the second involves the use of polydentate heteroaromatic ligands with a high rigidity and cyclometalating capability [10,13,16,18,21,22,23,24,25,26,27,28,29,30,31,32,33,34,35,36,37,38,39,40,41,42,43,44,45]. The strong bonding through their carbanionic donor atoms and the chelate effect provides a strong ligand field and, thus, energetically disfavours the thermal population of “dark” metal-centred (d−d*) excited states which lead to nonradiative decays. Heteroaromatic ligands also offer multiple options to tune the character of the excited triplet states, while involving a metal(d^8^)–ligand(π*) charge transfer (MLCT), intraligand (π−π*) charge transfer between donor and acceptor parts of the multidentate ligand(s) (IL or ILCT), or charge transfer between different ligands (L’LCT) [32,35,36,37,44,45,46]. Very often, the three last contributions are summarised as ligand-centred (LC), and the excited states of such complexes are frequently described as having a mixed ^3^LC/^3^MLCT character [10,14,15,16,17,18,22,23,24,35,36,37,41,45,46]. Thus, a number of Pt(II) complexes containing tri- or tetradentate multifunctional (*C*-bonding, *N*-bonding, further substituents) ligands were previously studied [10,18,21,22,23,24,25,26,27,28,29,30,31,32,33,34,35,36,37,38,39,40,41,42,43].

**Scheme 1:**
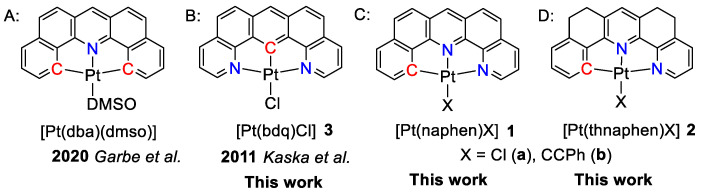
Schematic representation of the studied complexes and related work. A. [Pt(dba)(dmso)] [31]; B. [Pt(bdq)(Cl)] 3 [48]; C. [Pt(naphen)(X)] 1; D. [Pt(thnaphen)X] 2.

Amongst the tridentate heteroaromatic ligands, variations of the *C^N^N*, *N^C^N*, and *C^N^C* cores based on phenyl(*C*) and pyridyl(*N*) donor units have been reported [16,23,30,34,36,37,38,39,40,41,44,45,46]. In the prototypical systems with freely rotatable phenyl and pyridyl moieties, the double *C^N^C* cyclometalation in complexes of the dppy^2−^ ligand (H_2_dppy = 2,6-diphenylpyridine) has no marked benefit over the *C^N^N* (phbpy^−^) or *N^C^N* (dpb^−^) coordination in terms of the efficiency of the triplet photoluminescence (Hphbpy = 6-phenyl-2,2′-bipyridine; Hdpb = 2,6-di(2-pyridyl)benzene) [37]. On the other hand, the condensation of the three aromatic rings in the *C^N^C* system dba^2−^ (H_2_dba = dibenzo[*c,h*]acridine) (Scheme 1A) has led to markedly increased photoluminescence quantum yields (*Φ*_L_) for the complex [Pt(dba)(DMSO)] compared with the derivatives [Pt(dppy)(dmso)] [37]. The corresponding *N^C^N* derivative [Pt(bdq)(Cl)] (Hbdq = benzo[1,2-*h*:5,4-*h’*]diquinoline (Scheme 1B) was previously synthesised, but its luminescence properties had not been studied yet [47].

Thus, we embarked on a study using the fully fused *C^N^N* ligand naphen− (Hnaphen = naphtho[1,2-*b*][1,10]phenanthroline) in the Pt(II) complexes [Pt(naphen)(X)] (X = Cl (**1a**) or C≡CPh (**1b**)) (Scheme 1C). The *N^C^N* derivative [Pt(bdq)(Cl)] (**3**) was added to probe for the symmetry and the role of the position of the carbanionic *C* atom. To evaluate the impact of the rigidity of the ligand scaffold, we also used the hydrogenated derivative of naphen, namely thnaphen (Hthnaphen = 5,6,8,9-tetrahydro-naphtho[1,2-*b*][1,10]phenanthroline) and studied the two complexes [Pt(thnaphen)(X)] (X = Cl (**2a**) or C≡CPh (**2b**)) (Scheme 1D).

## 2. Results and Discussion

### 2.1. Preparation and Analytical Characterisation

**Scheme 2:**
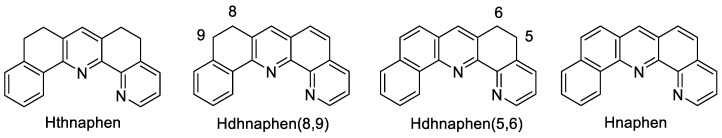
Schematic representation of the protoligands (protonated ligand precursors).

The protoligand 5,6,8,9-tetrahydro-naphtho[1,2-*b*][1,10]phenanthroline (Hthnaphen) (Scheme 2) represents a natural product which was isolated from *Cardiospermum halicacabum* [49]. We accomplished its synthesis following Risch’s original protocol [50] and obtained Hthnaphen with a 71% yield after column chromatographic purification (for details, see Materials and Methods Section 3.2 to Section 3.4). The subsequent dehydrogenation afforded the two isomeric dihydro-naphtho[1,2-*b*][1,10]phenanthrolines Hdhnaphen(8,9) and Hdhanaphen(5,6) (Scheme 2) and, finally, naphen with a yield of 61%. Alternatively, Hdhnaphen(8,9) was synthesised by Friedländer condensation from 8-amino-7-quinolinecarbaldehyde and 1-tetralone, as previously reported, with a 48% yield [51], and then dehydrogenated to Hnaphen with a yield of 67%.

The protoligands Hnaphen, Hthnaphen, and Hbdq were reacted with K_2_[PtCl_4_] in order to obtain the cyclometalated complexes in good to excellent yields [Pt(naphen)(Cl)] (**1a**) (78%), [Pt(thnaphen)(Cl)] (**2a**) (93%), and [Pt(bdq)(Cl)] (**3**) (57%). The analytical data of **3** were obtained as previously reported [47]. The Pt−Cl complexes **1a** and **2a** were then converted to the Pt−C≡CPh complexes [Pt(naphen)(C≡CPh)] (**1b**) (99%) and [Pt(thnaphen)(C≡CPh)] (**2b**) (90%) in excellent yields. The NMR spectra and MS of all of the new materials can be found in the Supplementary Material (Figures S1–S14).

### 2.2. Electrochemistry

**Figure 1:**
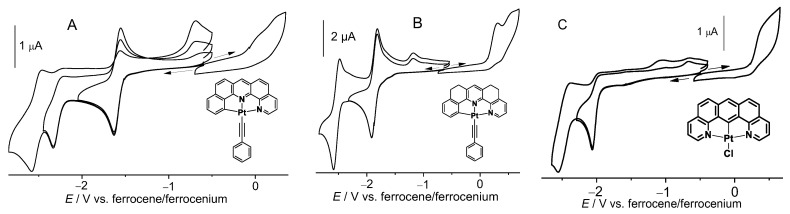
Cyclic voltammograms of **1b** (**A**), **2b** (**B**), and **3** (**C**) in 0.1 M *n*-Bu_4_NPF_6_/THF.

The two Pt(II) naphen complexes **1a** and **1b** showed a first reversible reduction at around −1.58 V, while for the thnaphen derivatives **2a** and **2b**, this wave shifted to −1.86 V (Figure 1, Table 1, more plots in Figures S15–S19). The second reduction waves were irreversible for the chlorido complexes and at least partially reversible for the Pt−C≡CPh derivatives **1b** and **2b**. The potential for the second waves was again lower (more negative) for **2b**, but interestingly, it was fully reversible for this complex, while for **1b**, the second wave was only partially reversible under the same conditions (Figure 1). When comparing **1a** and **2a** with the *C^N^C* derivative [Pt(dba)(dmso)] or the nonfused derivatives [Pt(phbpy)(Cl)] (*C^N^N*) and [Pt(dpb)(Cl)] (**3**) (*N^C^N*), the reduction potential increased (became less negative) along the series dpb < dba < bdq < thnaphen < phbpy < naphen (Table 1) in line with the assumption that *N^N*-containing ligands contain far better acceptor units of the bipyridine or phenanthroline type [23,51].

The reductions of the *N^C^N* complex [Pt(bdq)(Cl)] (**3**) and the *C^N^C*-coordinated [Pt(dba)(dmso)] were both markedly lower (more negative) and completely irreversible. When comparing these fully fused *C^N^N* (naphen) and *N^C^N* (bdq) complexes with those of the nonfused derivatives [Pt(phbpy)(Cl)] and [Pt(dpb)(Cl)] (Hphbpy = 6-phenyl-2,2′-bipyridine; Hdpb = 2,6-di(2-pyridyl)benzene), we found that also for the latter the symmetric derivative [Pt(dpb)(Cl)] showed an irreversible reduction behaviour. Remarkably, the 3,5-dimethylated derivative [Pt(Me_2_dpb)(Cl)] showed reversible reduction waves [48].

The oxidation processes were all irreversible and 0.35 V for the four naphen and thnaphen complexes, with the complexes containing the Cl^−^ coligand somewhat higher vs. C≡CPh. The value of the symmetric complex **3** was slightly higher and as expected from the doubly anionic character of the dba ligand, the potential of the complex [Pt(dba)(dmso)] was far higher. Interestingly, for the nonfused ligands, the potential of the *N^C^N* (dpb) complex was lower than the *C^N^N* (phbpy) derivative which is in line with the two pyridyl groups lowering the σ-donation power of the carbanionic phenyl core in dpb.

The electrochemical band gaps Δ*E* = Ox1 − Red1 were thus very different and increased from 1.89 eV [Pt(naphen)(C≡CPh)] (**1b**) to 2.19 for [Pt(thnaphen)(Cl)] (**2a**) within the *C^N^N* series of the naphen and thnaphen complexes. They were markedly exceeded by the *N^C^N* coordinated bdq derivative (2.50 eV) and the *C^N^C* complex [Pt(dba)(dmso)] (2.91 eV) (Table 1). Overall, the band gaps decreased along the series dba > dpb~bdq > phbpy > thnaphen > naphen, and the C≡CPh coligands markedly lowered them compared with the Cl derivatives for the thnaphen and naphen complexes.

The DFT-calculated contributions to the highest occupied molecular orbital (HOMO) and lowest unoccupied molecular orbital (LUMO) showed the expected higher symmetry for **3** compared with **1a** (Figure S20), but for both complexes, we found the assumed essentially Pt-based HOMO with large contributions of the Cl coligand. Only for **3**, a marked ligand contribution was found in the central phenyl core. The ligand-based LUMO of **3** was completely symmetric and distributed over the entire bdq^−^ ligand, while for **1a**, the LUMO was centred at the phenanthroline core, as expected. Although these calculations confirmed the preliminary assignment of the redox processes, they failed to explain the significant difference between **1a** and **3** concerning their reversibility.

### 2.3. UV-Vis Absorption Spectroscopy

**Figure 2:**
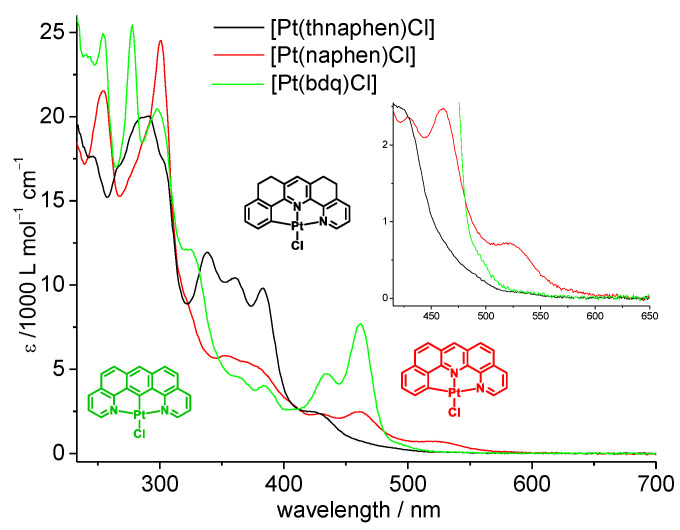
UV-Vis absorption spectra (molar absorption coefficients as a function of wavelength) of **1a**, **2a**, and **3** in CH_2_Cl_2_. Validity range: *c* = 1 × 10^−6^ M to 1 × 10^−4^ M in CH_2_Cl_2_ at 298 K.

The four complexes containing the *C^N^N* ligands naphen and thnaphen as well as X = Cl or C≡CPh coligands showed intense and structured band progressions from 220 to 300 nm (Figure 2, Figures S22 and S26, full data in Table S1) and partially structured bands in the range from 300 to 400 nm with markedly lower intensity. Very similar bands were also observed for the protoligands (Figure S21), and they can be assigned to transitions into π−π* states for both the protoligands and complexes. For the complexes, additional long-wavelength broad bands had their maxima in the 400 to 500 nm range (Table 2) and tailed down to cut-off values (i.e., where the absorption spectra reached the baseline) of 550 to 600 nm. The naphen complexes **1a** and **1b** showed more intense features at low energy, and the optical cut-offs were markedly red-shifted compared with the thnaphen derivatives **2a** and **2b**. The two C≡CPh complexes **1b** and **2b** similar cut-off energies as the Cl derivatives, but much higher intensities of the bands dominating the 400 to 500 nm range. The band energies or the symmetric *N^C^N* complex **3** were not very different from those of the naphen or thnaphen derivatives **1a** and **2a**, but the relative intensities deviated largely. In particular, the very intense band system peaking at 461 nm for **3** had equivalents for the unsymmetric complexes of only fractional intensity (Figure 2, Table 2). However, the optical cut-off was markedly higher (578 nm) and was very similar to the value found for the *C^N^C* complex [Pt(dba)(dmso)].

The optical band gaps derived from the cut-offs were slightly higher for the naphen and thnaphen complexes than the electrochemical band gaps, which represents the difference between the vertical Franck–Condon excitation and the geometrically “relaxed” redox states. Remarkably, the difference was very small for the thnaphen complexes **2a** and **2b**. For the two symmetric complexes **3** and [Pt(dba)(dmso)], the electrochemical band gaps were higher than the optical values. This is quite unexpected for the above-discussed reasons but agrees quite well with the irreversible character of the electrochemical reduction, which probably led to excessively negative values.

### 2.4. Steady-State Photoluminescence Spectroscopy

**Figure 3:**
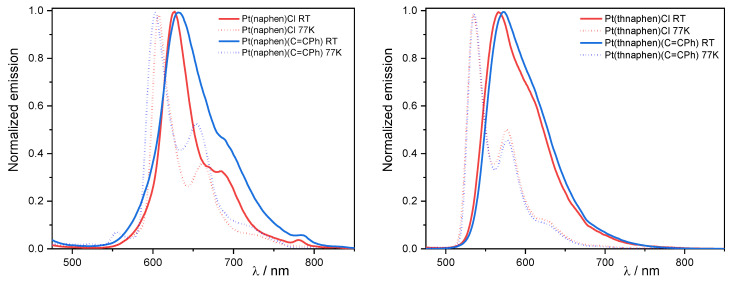
Left: Normalised photoluminescence spectra of [Pt(naphen)(Cl)] (**1a**) and [Pt(naphen)(C≡CPh)] (**1b**) in fluid Ar-purged CH_2_Cl_2_ solution at 298 K and in a frozen glassy matrix (CH_2_Cl_2_:MeOH 1:1) at 77 K. Right: Normalized photoluminescence spectra of **1a** and **1b** in fluid Ar-purged CH_2_Cl_2_ solution at 298 K and in a frozen glassy matrix (CH_2_Cl_2_:MeOH 1:1) at 77 K (*λ*_ex_ = 350 nm). In all cases, *c* = 1 × 10^−5^ M.

The complex **1a** showed a partially structured photoluminescence peaking at 628 nm when excited at 350 nm in fluid CH_2_Cl_2_ at 298 K (Figure 3, Table 3). For the C≡CPh derivative **1b**, while the maximum was only slightly red-shifted (632 nm), the vibrational progression was less pronounced (Figure 3). Interestingly, the symmetric isomer **3** showed an even more pronounced vibronic structure, and it was also blue-shifted (586 nm) when compared with **1a** (Figures S24 and S25). The two analogous thnaphen complexes **2a** and **2b** were markedly blue-shifted and showed poorly structured emission profiles with an almost identical maximum peaking at around 570 nm when compared with the naphen analogues. For the complexes [Pt(phbpy)(X)] containing the flexible *C^N^N* ligand phbpy^−^, marked differences were found for X = Cl vs. C≡CPh [53,54,55]. [Pt(phbpy)(Cl)] emitted at 565 nm at 298 K [54], whereas [Pt(phbpy)(C≡CPh)] showed an emission maximum at 582 nm [55] (in CH_2_Cl_2_). Both spectra were markedly blue-shifted with respect to the naphen complexes.

The differently pronounced vibrational progression of the emission spectra was mostly due to the varying contributions of ^3^MLCT or ^3^LC character [8,9,10,46,53,56,57]. In the case of the naphen complexes **1a** and **1b**, the LC character was more prominent compared to the thnaphen derivatives **2a** and **2b** and thus, a more defined vibronic progression was observed. The *C^N^C* derivative [Pt(dba)(dmso)] emitted at 588 nm [37], thus markedly blue-shifted compared to the naphen complex. Thus, it is possible to correlate the emission wavelength with the donor strength of the ligand: naphen^−^ < dba^2−^ ≤ bdq < thnaphen^−^. Compared to 298 K, the emission of the naphen and thnaphen complexes in the frozen glassy matrices (CH_2_Cl_2_:MeOH 1:1) revealed a marked blue-shift (30–40 nm), which was essentially due to a weaker charge transfer stabilization by restricted solvent reorientation at 77 K, hence decreasing the ^3^MLCT character of the emissive state. This led to an enhanced vibrational progression, which was also due to the reduced density of the solvent-related roto-vibrational states. The photoluminescence quantum yields *Φ*_L_ between 0.06 and 0.14 at room temperature for the naphen and thnaphen complexes were massively increased to 0.95 at 77 K. In addition, **3** showed only a very small blue-shift (7 nm) upon cooling. It also had already shown a quite high *Φ*_L_ at 298 K, which might be in part related to the massive blue-shift of the emission and can thus be explained by the energy gap law. On the other hand, besides the pronounced vibronic structure at both 298 and 77 K, the small shift upon cooling and the high *Φ*_L_ were in line with a high LC contribution to the emitting state.

In air-equilibrated solutions, the *Φ*_L_ of all of the complexes was below 0.02 and drastically increased in Ar-purged solutions, which is indicative of a triplet emission. In addition, the photoluminescence lifetimes (*τ*) significantly increased when going from air-equilibrated (250 to 530 ns) to Ar-purged solutions (1510 to 39,930 ns) at 298 K with the *N^C^N* complex **3** far above the *C^N^N* derivatives. At 77 K, they were in the µs range of 7 to 20 µs for the naphen and thnaphen complexes and a remarkable 46 µs for **3**.

For the [Pt(phbpy)(C≡CPh)] complex containing the more flexible phbpy ligand, a pronounced blue-shift of about 40 nm was reported when going from 298 K (CH_2_Cl_2_) to the frozen glassy matrix of MeOH/EtOH at 77 K (582 to 540 nm) alongside an increased vibrational progression [55], very similar to what we observed for the naphen and thnaphen complexes **1a**, **1b**, **2a**, and **2b**. In addition, the *Φ*_L_ of the two C≡CPh complexes were only marginally higher (0.06 and 0.14) than those of the phbpy derivative (0.04). Thus, the increasing rigidity along the series phbpy < thnaphen < naphen had no large effect on the *Φ*_L_. However, the lifetimes at 298 K (around 4 and 1.5 µs) were much shorter for the phbpy complex (0.4 µs), thus fitting nicely to this series. For the chlorido complex [Pt(phbpy)(Cl)], the emission maximum also shifted to higher energy when going from 298 K (CH_2_Cl_2_) to 77 K (MeOH/EtOH frozen glassy matrix) (565 to 540 nm) alongside a pronounced vibronic structure at 77 K [54]. So, for both the Pt Cl and C≡CPh complexes of the flexible phbpy^−^ ligand, the character of the excited state shifted towards a higher ^3^LC character, in quite a similar way as we found for the naphen derivatives. Unfortunately, the *Φ*_L_ for the phbpy complexes have not been reported, so we can only state that the rigidification of the ligands along the series phbpy < thnaphen < naphen significantly increased the lifetimes of both the Cl and C≡CPh complexes. All three Pt C≡CPh complexes showed broad emission profiles at 298 K indicative of a large ^3^MLCT contribution and narrowed emission at 77 K, in line with an increased ^3^LC character. So, at 298 K, the L’LCT contributions from the C≡CPh ligand seemed to not be particularly pronounced.

For complex **3**, the effect of the rigidification was already obvious from the notorious red-shift of the emission band at 586 nm (298 K) when compared to the [Pt(dpb)(Cl)] derivative containing the flexible dipyridyl-benzene ligand that emits at 491 nm [52]. The vibrational structure of the emission and thus, the high ^3^LC character of the emitting state seemed to be similar. The lifetime of [Pt(dpb)(Cl)] at 77 K (glassy diethyl ether/isopentane/EtOH 2:2:1) was reported as 7.0 µs [57]. Thus, the rigidity of the bdq ligand compared to the flexible dbp ligand in the isoleptic complexes significantly enhanced the lifetime to 45 µs.

As shown in Figures S24 to S29, minimal traces of the metal-free ligands were detected. It is worth mentioning that as the fluorescence quantum yield (*Φ*_F_ of the ligands (Hnaphen, Hthnaphen, and Hbdq) alone was ≈40%, even negligible trace amounts could be detected by modern photoluminescence spectrometers (a trace amount below 0.01% of a highly fluorescent species can be detected, even if the main yet weak triplet emitter is pure according to usual standards, including NMR and elemental microanalysis, where our compounds showed a purity > 99%). However, this minimal ligand contribution did not practically affect the *Φ*_L_ of the complexes (as confirmed when processing the emission spectra on the integrating sphere, no changes were detected whether the ligand fluorescence was included or not) or the lifetimes (as shown in Table 3, at RT, all the decays were monoexponential).

### 2.5. Time-Resolved Emission and Transient Absorption Spectroscopy on [Pt(naphen)(Cl)] (***1a***)

**Figure 4:**
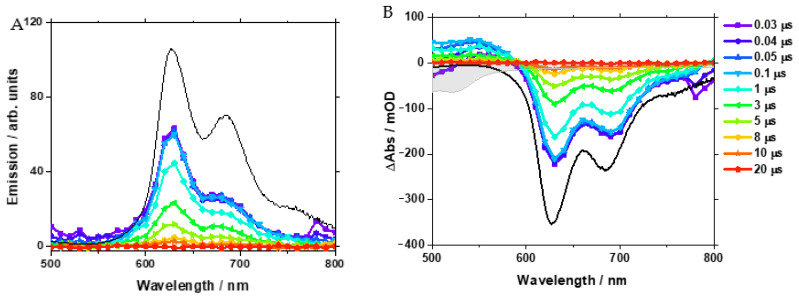
Nanosecond time-resolved emission spectra (**A**) and transient absorption spectra (**B**) of [Pt(naphen)(Cl)] (**1a**) at selected time points. The black lines represent the steady-state emission spectrum (**A**) and the inverted steady-state emission spectrum (**B**). In panel (**B**), the grey-shaded spectrum indicates the inverted ground-state spectrum of the complex.

Furthermore, nanosecond time-resolved emission and transient absorption (TA) spectroscopy were carried out. For these experiments, **1a** was dissolved in CH_2_Cl_2_ under an N_2_ atmosphere, and the sample was excited at 355 nm. Figure 4 shows the transient emission spectra at selected time points, which spectrally matched with the steady-state emission spectrum with its structured shape showing maxima at 630 and 680 nm (Figure 4A).

The shoulder in the steady-state emission at about 760 nm was not observed in the transient spectra, most likely due to the limited signal-to-noise ratio in the ns time-resolved experiment. The absence of spectral shifts in the time range probed indicates that any excited state of relaxation affecting the emissive state was completed within 30 ns. The kinetic analysis of the time-resolved emission data yielded a lifetime of 3 µs (Figure S45).

In addition, the ns TA spectra (Figure 4B) were dominated by the contributions of the emission, which were accompanied by a rather weak excited-state absorption (ESA) in the range from 500 to 590 nm. Both the ESA and emission features were found to decay with the same time constant of 3 µs (see also Figure S45). The concerted decay of the long-lived emission and ESA signals indicates that the emission stems from the phosphorescence of the mixed ^3^LC/^3^MLCT state of the complex. Due to the limited time resolution in the ns experiments, we did not observe the features of the ^1^LC/^1^MLCT, which apparently relaxed within the temporal resolution of our experiment via ISC to the ^3^LC /^3^MLCT state.

## 3. Materials and Methods

### 3.1. Materials

First, 2-((dimethylamino)methyl)-3,4-dihydronaphthalen-1(2*H*)-one hydrochloride was prepared previously as reported [50]. The complex [Pt(bdq)(Cl)] (**3**) and the Hbdq ligand were prepared as previously described [47] and correctly analysed.

### 3.2. Preparation of the Protoligand 5,6,8,9-Tetrahydronaphtho[1,2-*b*][1,10]phenanthroline (Hthnaphen)

Under inert conditions, a solution of 0.74 g 6,7-dihydroquinolin-8(5*H*)-one (5.0 mmol, 1 equivalent) in 20 mL DMF was heated to 160 °C. Subsequently, a suspension of 1.44 g 2-((dimethylamino)methyl)-3,4-dihydronaphthalen-1(2*H*)-one hydrochloride (6.0 mmol, 1.2 equivalents.) and 2.00 g NH_4_OAc (26 mmol) in 15 mL DMF was added dropwise within 45 min. Stirring at 160 °C was continued for 2 h, and, after cooling to room temperature, the solvent was removed in vacuo. The sticky residue was treated with 15 mL water, alkalised by adding dilute aq. NaOH and extracted with CH_2_Cl_2_ (4 × 25 mL). The combined organic extracts were neutralised by washing with water and dried over Na_2_SO_4_. The solvent was removed under reduced pressure, and the residue was purified by column chromatography (silica, CH_2_Cl_2_/acetone = 10/1). Finally, the oily crude product was washed with *n*-hexane (2 × 5 mL) to give a grey-yellow solid. Yield: 1.01 g (3.55 mmol, 71%). ^1^H NMR (500 MHz, CDCl_3_): *δ* = 8.74 (dd, 1H, *J* = 1.7, 7.6 Hz), 8.62 (dd, 1H, *J* = 1.3, 7.7 Hz), 7.54 (dd, 1H, *J* = 1.7, 7.6 Hz), 7.41–7.19 (m, 4H), 7.21 (dd, 1H, *J* = 4.8, 7.3 Hz), 2.97 (m, 4H), 2.93 (m, 4H) ppm; HR-ESI-MS (+) *m/z* = 285.13922 [M+H]^+^ (calc. 285.13918).

### 3.3. Preparation of the Protoligand Naphtho[1,2-*b*][1,10]phenanthroline (Hnaphen)

Under ambient conditions, 185 mg Hthnaphen (0.65 mmol, 1 equivalents.) and 100 mg Pt/C (10%) in 4 mL nitrobenzene were heated up to 210 °C. After four days, additional 50 mg Pt/C (10%) was added, and the reaction mixture was heated up to 210 °C for another two days. Then, the mixture was filtered through Celite and rinsed three times with a mixture of CH_2_Cl_2_/MeOH (10:1). The solvent was removed under reduced pressure, and the raw product was purified by column chromatography (silica, gradient CHCl_3_ → CHCl_3_ + 2%MeOH). The product was obtained as an off-white solid. Yield: 111 mg (0.40 mmol, 61%). ^1^H NMR (300 MHz, CDCl_3_): *δ* = 9.90 (d, *J* = 7.8 Hz, 1H), 9.32 (dd, 1H, *J* = 1.6, 4.4 Hz), 8.71 (s, 1H), 8.29 (dd, 1H, *J* = 8.0, 1.6 Hz), 7.95 (t, 2H, *J* = 8.2 Hz), 7.88–7.75 (m, 5H), 7.70 (dd, 1H, *J* = 4.4, 8.0 Hz) ppm; HR-ESI-MS (+) *m/z* = 281.10751 [M+H]^+^ (calc. 281.10732).

### 3.4. Syntheses of the Complexes [Pt(C^N^N)(Cl)]—General Description

The corresponding ligand and 208 mg K_2_[PtCl_4_] (0.5 mmol) were suspended in glacial acetic acid (60 mL). The suspension was heated up to 110 °C for three days. Formation of a precipitate was observed over time. After cooling down to room temperature, the precipitate was filtered off and washed consecutively with acetic acid, water, and diethyl ether. From the aqueous phase, unreacted K_2_[PtCl_4_] was recovered. The crude precipitated products were carefully recrystallised from mixtures of CH_2_Cl_2_/diethyl ether (*v:v* = 2:1).

#### 3.4.1. [Pt(naphen)(Cl)] (**1a**)

From 80 mg Hnaphen (0.29 mmol, 1 equivalent) and 144 mg K_2_[PtCl_4_] (0.35 mmol, 1.2 equivalents); red solid. Yield: 115 mg (0.23 mmol, 78%). Elemental anal. found (calcd. for): C_20_H_11_ClN_2_Pt (509.85): C 47.11 (47.12), H 2.20 (2.17), N 5.48 (5.49). ^1^H NMR (600 MHz, CD_2_Cl_2_): *δ =* 9.14 (dd, 1H, *J* = 4.9, 1.3 Hz), 8.79 (s, 1H), 8.55 (d, 1H, *J* = 8.2 Hz), 8.03 (d, 2H, *J* = 9.0 Hz), 7.97 (dd, 1H, *J* = 4.9, 8.2 Hz), 7.85–7.82 (m, 2H), 7.79 (d, 1H, *J* = 7.0 Hz, *J*_Pt-H_ = 49 Hz,), 7.70 (d, 1H, *J* = 8.9 Hz), 7.65–7.60 (m, 2H) ppm; EI-MS(+) (70 eV) *m/z* = 510 [M]^+^, 473 [M-Cl]^+^, 280 [Hnaphen]^+^.

#### 3.4.2. [Pt(thnaphen)(Cl)] (**2a**)

From 28 mg Hthnaphen (0.1 mmol, 1 equivalent) and 50 mg K_2_[PtCl_4_] (0.12 mmol, 1.2 equivalents); orange solid. Yield: 48 mg (0.093 mmol, 93%). Elemental anal. found (calcd. for): C_20_H_15_ClN_2_Pt (513.88): C 46.77 (46.75), H 2.90 (2.94), N 5.45 (5.45). ^1^H NMR (600 MHz, CD_2_Cl_2_): *δ =* 8.61 (d, 1H, *J* = 5.0 Hz, H6’), 7.76 (d, 1H, *J* = 7.7 Hz; H4’), 7.50 (dd, 1H, *J* = 7.7, 5.3 Hz, H5’), 7.35 (s, 1H, H4), 7.23 (d, 1H, *J*_Pt-H_ = 44 Hz, *J* = 7.5 Hz, Hb), 7.08 (t, 1H, *J* = 7.5 Hz, Hc), 6.81 (d, 1H, *J* = 7.5 Hz, Hd), 3.18 (t, 2H, *J* = 7.7 Hz, CH_2_), 3.10 (t, 2H, *J* = 7.6 Hz, CH_2_), 2.99 (m, 4H, CH_2_) ppm. EI-MS(+) (70 eV) *m/z* = 514 [M]^+^, 478 [M-Cl]^+^, 476 [M-Cl-H_2_]; ESI-MS(+): *m/z* = 590.047 [M+Cl+CH_3_CN]^+^ (calc. 590.051), 519.115 [M-Cl+CH_3_CN]^+^ (calc. 519.115), 285 [Hthnaphen]^+^_._

### 3.5. Syntheses of the Complexes [Pt(C^N^N)(C≡CPh)]—General Description

The chlorido complexes [Pt(*C^N^N*)(Cl)] were dissolved in degassed CH_2_Cl_2_. Phenylacetylene, CuI (8 mol%) and NEt_3_ were added. The reaction mixture was stirred at room temperature overnight in the absence of light. The resulting dark solution was treated with diethyl ether until no further solid precipitated. The precipitate was filtered off and thoroughly washed with diethyl ether and water. Optionally, the product can be recrystallised from CH_2_Cl_2_ and diethyl ether. The products were recrystallised from mixtures of CH_2_Cl_2_ and diethyl ether (*v:v* = 1:2).

#### 3.5.1. [Pt(naphen)(C≡CPh)] (**1b**)

From 70 mg **1a** (0.135 mmol), 45 μL phenylacetylene (0.411 mmol), 2.1 mg CuI (0.011 mmol), 1.3 mL NEt_3_ in 50 mL CH_2_Cl_2_; orange solid. Yield: 78 mg (0.135 mmol, 99%). Elemental anal. found (calcd. for): C_28_H_16_N_2_Pt (575.52): C 58.44 (58.43), H 2.80 (2.80), N 4.85 (4.87). ^1^H NMR (300 MHz, CD_2_Cl_2_): *δ* = 9.19 (d, *J* = 3.9 Hz, *J*_PtH_ = 18 Hz, 1H), 8.67 (s, 1H), 8.46 (d, *J* = 7.3 Hz, 1H), 7.93 (t, *J* = 8.2 Hz, 2H), 7.83–7.74 (m, 3 H), 7.65–7.55 (m, 4 H), 7.33 (t, *J* = 7.5 Hz, 2H), 7.21 (t, *J* = 7.4 Hz, 1H) ppm; EI-MS(+) (70 eV) *m/z* = 575 [M]^+^, 284 [Hnaphen]^+^.

#### 3.5.2. [Pt(thnaphen)(C≡CPh)] (**2b**)

From 51.4 mg **2a** (0.1 mmol), 32 μL phenylacetylene (0.292 mmol), 1.5 mg CuI (0.008 mmol), 0.8 mL NEt_3_ in 50 mL CH_2_Cl_2_; orange solid. Yield: 52 mg (0.09 mmol, 90%). Elemental anal. found (calcd. for): C_28_H_20_N_2_Pt (579.55): C 58.08 (58.03), H 3.50 (3.48), N 4.81 (4.83). ^1^H NMR (300 MHz, CD_2_Cl_2_): *δ* = 8.64 (d, 1H, *J* = 5.4 Hz, *J*_PtH_ = 18 Hz), 7.67 (d, 1H, *J* = 7.7 Hz), 7.46 (d, 2H, *J* = 7.3 Hz), 7.37–7.25 (m, 4H), 7.16 (t, 1H, *J* = 7.4 Hz), 7.03 (t, 1H, *J* = 7.6 Hz), 6.78 (d, 1H, *J* = 7.4 Hz), 3.18 (d, 2H, *J* = 6.7 Hz), 3.11 (d, 2H, *J* = 6.7 Hz), 2.98–2.95 (m, 4H) ppm; EI-MS(+) (70 eV) *m/z* = 579 [M]^+^, 284 [Hthnaphen]^+^.

### 3.6. Instrumentation

^1^H, ^13^C and correlation spectra were recorded on a *Bruker* Avance II 300 MHz spectrometer equipped with a double resonance (BBFO) 5 mm observe probe head with z-gradient coil, a *Bruker* Avance III 499 MHz spectrometer with a TCI Prodigy 5 mm probe head with z-gradient coil (^1^H/^19^F ^13^C ^15^N ^2^H), or a *Bruker* Avance II+ 600 MHz spectrometer with a L.T. TBI 5 mm probe head with z-gradient coil (^1^H ^31^P X ^2^H) (all Bruker, Rheinhausen, Germany). ^1^H and ^13^C chemical shifts were reported relative to tetramethylsilane (TMS). UV–vis absorption spectra were recorded on a *Varian* 50 Scan spectrophotometer. Elemental analyses were obtained using a *HEKAtech* CHNS EuroEA 3000 analyser (HEKAtech, Wegberg, Germany). EI-MS spectra in the positive mode were measured using a *Finnigan* MAT 95 mass spectrometer (Thermo Finnigan Mat, Bremen, Germany). HR-ESI-MS(+) spectra were measured using a *Thermo Scientific* LTQ Orbitrap XL mass spectrometer via electron spray ionisation and a FTMS analyser (ThermoFisher Scientific, Waltham, MA, USA). Simulations were performed using ISOPRO 3.0 (Mike Senko, Sunnyvale, CA, USA). Electrochemical measurements were carried out in 0.1 M *n*-Bu_4_NPF_6_ solution in THF or CH_2_Cl_2_ at 298 K and at 100 mV/s scan rate if not stated otherwise, using a three-electrode configuration (glassy carbon working electrode, Pt counter electrode, Ag/AgCl pseudo reference electrode), and a *Metrohm* Autolab PGSTAT30 or µStat400 potentiostat (Metrohm, Filderstadt, Germany). The potentials were referenced against the ferrocene/ferrocenium redox couple as internal standard.

### 3.7. Photophysical Measurements

Steady-state excitation and emission spectra were recorded on a FluoTime 300 spectrometer from PicoQuant (Berlin, Germany) equipped with a 300 W ozone-free Xe lamp (250–900 nm), a 10 W Xe flash-lamp (250−900 nm, pulse width ca. 1 µs) with repetition rates of 0.1–300 Hz, double excitation monochromators (Czerny–Turner type, grating with 1200 g/mm, blaze wavelength: 300 nm), diode lasers (pulse width < 80 ps) operated by a computer-controlled laser driver PDL-828 “Sepia II” (repetition rate up to 80 MHz, burst mode for slow and weak decays), two double-grating emission monochromators (Czerny–Turner, selectable gratings blazed at 500 nm with 2.7 nm/mm dispersion and 1200 grooves/mm or blazed at 1200 nm with 5.4 nm/mm dispersion and 600 grooves/mm) with adjustable slit width between 25 µm and 7 mm, and Glan–Thompson polarisers for excitation (Xe-lamps) and emission. Different sample holders (Peltier cooled sample mounting unit ranging from −15 to 110 °C and adjustable front face sample holder). Two detectors, namely a PMA Hybrid-07 (transit time spread FWHM < 50 ps, 200–850 nm) and a H10330C-45-C3 NIR detector (transit time spread FWHM 0.4 ns, 950–1700 nm) from Hamamatsu (Hamamatsu Photonics, Ltd., Shizuoka, Japan). Steady-state and fluorescence lifetimes were recorded in TCSPC mode by a PicoHarp 300 (minimum base resolution 4 ps) or in MSC mode by a TimeHarp 260, where up to several ms can be detected. Emission and excitation spectra were corrected for source intensity (lamp and grating) by standard correction curves. Lifetime analysis was performed using the commercial EasyTau 2 software (PicoQuant). The quality of the fit was assessed by minimising the reduced chi-squared function (χ^2^) and visual inspection of the weighted residuals and their autocorrelation. Luminescence quantum yields were measured with a Hamamatsu Photonics absolute PL quantum yield measurement system (C9920-02) equipped with a L9799-01 CW Xenon light source (150 W), monochromator, C7473 photonic multichannel analyser, and integrating sphere and by employing U6039-05 PLQY measurement software (Hamamatsu Photonics). All cuvettes used were round quartz cuvettes, and the solvents were of spectrometric grade (Uvasol^®^, Merck, Darmstadt, Germany).

### 3.8. Nanosecond Time-Resolved Emission/Transient Absorption

Nanosecond emission/transient absorption spectra were collected to study the long-lived excited state of the complex [58]. In detail, pump pulses centred at 355 nm were generated using a Continuum Surelite Nd:YAG laser (Soliton, Gilching, Germany) with a pulse duration of 5 ns and a pulse-to-pulse repetition rate of 10 Hz. A 75 W xenon arc lamp provided the probe light. Spherical concave mirrors were used to focus the probe light into the sample and to refocus the light on the entrance slit of a monochromator (Acton, Princeton Instruments, Acton, MA, USA). The probe light was detected by a Hamamatsu R928 photomultiplier tube (Hamamatsu Photonics) mounted on a five-stage base at the monochromator exit slit, and the signal was processed by a commercially available detection system (Pascher Instruments AB, Lund, Sweden). By switching off the probe light, emission decay could be detected with ns-temporal resolution with the same set-up [59]. The initial signals, i.e., up to 30 ns after photoexcitation, tend to result in contributions from the experimental response function and are hence not considered. The samples were prepared under inert conditions in degassed CH_2_Cl_2_ by performing several freeze–pump–thaw cycles. The OD of the samples at the excitation wavelength was around 0.35 in a 1 cm cuvette. For all measurements, the pump power was fixed at 0.4 mJ.

### 3.9. DFT Calculations

Quantum chemical calculation based on the density functional theory (DFT) were carried out using the so-called “resolution of identity“ Coulomb approximation [60,61], which was implemented in the program package TURBOMOLE [62] under the TMoleX [63] platform. Molecular structures were first geometry-optimised using the hybrid functional B3LYP [64,65,66] and the double-ξ valence basis set def-SV(P) [67]. Further optimisations used the triple-ξ valence basis set def2-TZVP [68] for C, H, N, O, and LAN-L2DZ for Pt using effective core potentials (ECP) (n−1) by Hay and Wadt [69,70,71].

## 4. Conclusions

In this work, we were able to compare the tridentate *C^N^N*-coordinated and fully condensated cyclometalating ligand naphen^−^ (Hnaphen = naphtho[1,2-*b*][1,10]phenanthroline) and its slightly more flexible tetrahydro-derivative thnaphen− (Hthnaphen = 5,6,8,9-tetrahydro-naphtho[1,2-*b*][1,10]phenanthroline) with the *N^C^N*-coordinating bdq^−^ chromophore (Hbdq = benzo[1,2-*h*:5,4-*h’*]diquinoline) in their corresponding Pt(II)X complexes (X = Cl (**1a**, **2a**, and **3**) or C≡CPh (**1b** and **2b**)) and the previously reported *C^N^C* ligand dba^2−^ (H_2_dba = dibenzo[*c,h*]acridine) in the corresponding complex [Pt(dba)(dmso)]. We synthesised the new naphen and thnaphen complexes and were able to reproduce the synthesis of the complex [Pt(bdq)(Cl)] (**3**) in very good yields.

The cyclic voltammetry showed reversible reductions for the *C^N^N* complexes, with markedly fewer negative potentials (around −1.6 V, vs. ferrocene) for the species containing the fully condensated naphen ligand (**1a** and **1b**) compared with the thnaphen (around −1.9 V) derivatives **2a** and **2b**. Together with the irreversible oxidations at around +0.3 V for all of the complexes, the naphen made a difference in the electrochemical gap of about 0.3 V (1.9 vs. 2.2). The bdq complex **3** with its *N^C^N* pattern was completely different. The irreversible reduction at around −2 V was comparable to the first reduction in the *C^N^C*-coordinated complex [Pt(dba)(dmso)]. Both ligand systems lacked a good electron acceptor as the phenanthroline unit in the *C^N^N* ligand naphen.

Long-wavelength UV-Vis absorption bands were found around 520 to 530 nm for the *C^N^N*-coordinated species, and the C≡CPh complexes **1b** and **2b** appeared red-shifted compared with the Cl derivatives **1a** and **2a**. The *C^N^C*-coordinated complex [Pt(dba)(dmso)] was at almost the same energy. The *N^C^N*-coordinated bdq complex was markedly blue-shifted (493 nm).

The steady-state photoluminescence spectra of the *C^N^N*-coordinated naphen and the thnaphen complexes at 298 K in solution showed poorly structured emission spectra peaking at around 630 nm and 570 nm for the thnaphen. The bdq complex showed a pronounced vibrational progression and an emission maximum at 586 nm, very similar to what has been reported for the *C^N^C* complex [Pt(dba)(dmso)] (588 nm). While the vibronic structure indicated a higher LC character for the *N^C^N* bdq and *C^N^C* dba complexes compared with the *C^N^N*-coordinated naphen and tmnaphen complexes, the blue-shift was a result of the different *N^C^N* or *C^N^C* vs. *C^N^N* coordination with the best acceptor unit *N^N* found exclusively in the naphen and thnaphen complexes. The photoluminescence quantum yields *Φ*_L_ significantly increased when going from Ar-purged solutions at 298 K (0.06 to 0.24) to frozen glassy matrices at 77 K (0.80 to 0.95), alongside prolonged lifetimes *τ* (few ns to 7.20 µs) for the naphen and thnaphen complexes. Due to their rigidity, they showed far longer lifetimes (~×10) than [Pt(phbpy)(X)] (X = Cl or C≡CPh), i.e., the derivatives containing the more flexible *C^N^N* ligand. For the rigid bdq and dba complexes, the *Φ*_L_ at 298 K was markedly higher (around 0.3) and the *τ* much longer (ca. 4 µs and 16 µs). At 77 K, the *Φ*_L_ were about 0.8 and the lifetimes more than 10 times longer. Thus, they outperformed the derivatives containing the more flexible ligands based on dipyridylbenzene and diphenyl-pyridine. The nanosecond time-resolved study on [Pt(naphen)(Cl)] (**1a**) showed a phosphorescence signal originating from a mixed ^3^LC/^3^MLCT excited state with an emission lifetime of 3 µs, in agreement with the steady-state data. The *C^N^N*-coordinated naphen and thnaphen complexes are interesting candidates for a red emission >600 nm, and their performance in various host materials will be studied in the near future. The symmetric *N^C^N*- or *C^N^C*-coordinated complexes emitted at wavelengths <600 nm and were already good emitters at 298 K in fluid solution. In addition, their potential for electro-optical devices such as OLEDs will be prospectively investigated.

## Figures and Tables

**Table 1:** Selected redox potentials of [Pt(L)(X)] and comparable complexes ^a^.

[Pt(L)(X)]	*E*_1/2_ Red2	*E*_1/2_ Red1	*E*_pa_ Ox1	Δ*E* = Ox1 − Red1
**1a**	−2.24 irr	−1.57	0.36	1.93
**1b**	−2.27	−1.59	0.30 ^b^	1.89
**2a**	−2.59 irr	−1.86	0.34	2.19
**2b**	−2.53	−1.86	0.30 ^c^	2.16
**3**	−2.55 irr	−2.06 irr	0.41	2.47
Other Pt complexes				
[Pt(dba)(dmso)] ^d^	−2.70 irr	−1.98 irr	0.93	2.91
[Pt(phbpy)(Cl)] ^e^	−2.48	−1.78	0.40	2.19
[Pt(dpb)(Cl)] ^e,f^	-	−2.14 irr	0.35	2.49
[Pt(Me_2_dpb)(Cl)] ^e^	−2.69	−2.24	0.35	2.59

^a^ From cyclic voltammetry in *n*-Bu_4_NPF_6_/THF. Potentials in V vs. ferrocene/ferrocenium: half-wave potentials *E*_1/2_ for (partially) reversible processes; cathodic peak potentials *E*_pc_ for irreversible reductions (irr); accuracy of potentials: ±0.003 V. ^b^ Second oxidation at 0.28 V. ^c^ Second oxidation at 0.31 V. ^d^ From Ref. [37]. ^e^ From Ref. [48]. ^f^ From Ref. [52], measured in MeCN.

**Table 2:** Long-wavelength UV-Vis absorptions of the [Pt(L)(X)] complexes ^a^.

[Pt(L)(X)]	*λ* (ε)	*λ* (ε)	*λ* (ε)	*λ* (ε)	Cut-Off ^b^		Echem Gap (eV) ^c^
					(nm)	(eV)	
**1a**	401 (3.4)	428 (2.6)	461 (2.7)	523 (0.9)	600	2.07	1.93
**1b**	390 (5.1)	433 (2.5)	464 (3.3)	534 (1.2)	606	2.05	1.89
**2a**	383 (9.8)	428 (2.3)	452 (1.0)	524 (0.1)	563	2.20	2.19
**2b**	387 (9.7)	430 (5.3)	451 (5.0)	533 (0.3)	563	2.20	2.16
**3**	384 (4.1)	433 (4.8)	461 (7.7)	493 (0.6)	568	2.18	2.47
[Pt(dba)(dmso)] ^d^	409 (3.9)	461 (1.0)	498 (1.8)	529 (2.3)	580	2.14	2.91

^a^ Absorption maxima *λ*_abs_ in nm (*ε* in 10^3^ M^−1^ cm^−1^) in CH_2_Cl_2_ (298 K). ^b^ The “cut-off” is defined as the wavelength of zero absorption and is obtained through extrapolation of the low-energy slope of the lowest energy absorption band. ^c^ Electrochemical gap = Δ*E* = Ox1 − Red1 (see Table 1). ^d^ From Ref. [37], measured in THF.

**Table 3:** Selected photophysical data of the Pt complexes ^a^.

	*λ*_em,max_	*τ*_(air, 298 K)_/ns	*τ*_(Ar, 298 K)_/ns	*τ*_(77 K)_/µs	*Φ*_L (air)_	*Φ*_L (Ar)_	*Φ*_L (77 K)_
**1a**	628 _(298 K)_ 604 _(77 K)_	*τ* = 517 ± 1	*τ* = 4955 ± 2	*τ*_1_ = 24.7 ± 0.6 (63%) *τ*_2_ = 13.8 ± 0.8 (37%) *τ*_av_amp_ = 20.57 ± 0.05	<0.02	0.08	0.90
**1b**	632 _(298 K)_ 602 _(77 K)_	*τ* = 452.6 ± 0.9	*τ* = 4146 ± 3	*τ*_1_ = 29.8 ± 0.5 (24%) *τ*_2_ = 16.38 ± 0.19 (76%) *τ*_av_amp_ = 19.64 ± 0.03	<0.02	0.06	0.80
**2a**	570 _(298 K)_ 530 _(77 K)_	*τ* = 394.0 ± 0.6	*τ* = 2205 ± 6	*τ*_1_ = 16.3 ± 0.3 (25%) *τ*_2_ = 10.14 ± 0.12 (75%) *τ*_av_amp_ = 11.637 ± 0.014	<0.02	0.24	0.95
**2b**	571 _(298 K)_ 531 _(77 K)_	*τ* = 253.2 ± 0.2	*τ* = 1512 ± 1	*τ*_1_ = 9.14 ±0.04 (13%) *τ*_2_ = 6.505 ± 0.006 (87%) *τ*_av_amp_ = 6.857 ± 0.002	<0.02	0.14	0.85
**3**	586 _(298 K)_ 581 _(77 K)_	*τ* = 534 ± 3	*τ* = 39,930 ± 30	*τ*_1_ = 65.6 ± 0.3 (55%) *τ*_2_ = 23.7 ± 0.3 (45%) *τ*_av_amp_ = 46.66 ± 0.08	<0.02	0.32	0.80

^a^ Obtained under different conditions: dilute air-equilibrated (air) or argon-purged samples (Ar) in fluid CH_2_Cl_2_ solution at RT or frozen glassy matrices of CH_2_Cl_2_:MeOH 1:1 at 77 K, irradiated at *λ*_ex_ = 350 nm. Emission maxima, *λ*_em,max_; photoluminescence quantum yields *Φ*_L_ (accuracy ± 0.02); lifetimes *τ*. For the biexponential decays at 77 K, the amplitude-weighted average lifetimes (*τ*_av_amp_) are shown. Raw time-resolved photoluminescence decays including individual fitting components and their relative amplitudes (for biexponential decays) are available in the ESI, Figures S30–S44.

## Data Availability

Data may be requested directly from the authors.

## Supplementary Materials

The following supporting information can be downloaded at https://www.mdpi.com/article/10.3390/molecules27207022/s1.

## Sample Availability

Samples of the compounds are available on request (A.K.).
