# Supplementary material for: Photophysical Study on the Rigid Pt(II) Complex [Pt(naphen)(Cl)] (Hnaphen = Naphtho[1,2-b][1,10]Phenanthroline and Derivatives
Source: Molecules. 2022 Oct 18;27(20):7022. doi: 10.3390/molecules27207022 (PMC9606891; doi:10.3390/molecules27207022)
Supplement: Supplementary file 1 [file molecules-27-07022-s001.zip › molecules-1943005-supplementary.pdf]

Supplementary Materials

Photophysical Study on the Rigid Pt(II) Complex [Pt(naphen)Cl] (Hnaphen = naphtho[1,2-*b*][1,10]phenanthroline and Derivatives

Maren Krause[1], Iván Maisuls[2], Stefan Buss[2], Cristian A. Strassert[2], Andreas Winter[3,4], Ulrich S. Schubert[3,4], Shruti S. Nair[5,6], Benjamin Dietzek-Ivansic[5,6]*, Axel Klein[1]*

[1] Universität zu Köln, Department für Chemie, Institut für Anorganische Chemie, Greinstraße 6, 50939 Köln, Germany.

[2] Westfälische Wilhelms-Universität Münster, Institut für Anorganische und Analytische Chemie, Corrensstr. 28/30, 48149 Münster, Germany; CeNTech, CiMIC, SoN, Heisenbergstr. 11, 48149 Münster, Germany.

[3] Friedrich Schiller University Jena, Laboratory of Organic and Macromolecular Chemistry (IOMC), Humboldtstr. 10, 07743 Jena, Germany.

[4] Friedrich Schiller University Jena, Center for Energy and Environmental Chemistry Jena (CEEC Jena), Philosophenweg 7a, 07743 Jena, Germany.

[5] Friedrich Schiller University Jena, Institute for Physical Chemistry (IPC), Helmholtzweg 4, 07743 Jena, Germany.

[6] Leibniz Institute for Photonic Technologies Jena (IPHT) Albert-Einstein-Str. 9, 07745 Jena, Germany.

contents:

Supporting Figures

Figure S1. 300 MHz ^{1}H NMR spectrum of 5,6,8,9-tetrahydro-naphtho[1,2-*b*][1,10]phenanthroline (Hthnaphen) in CD_2Cl_2.
Figure S2. 300 MHz ^{1}H NMR spectrum of naphtho[1,2-*b*][1,10]phenanthroline (Hnaphen) in $CDCl_3$.
Figure S3. 300 MHz ^{1}H NMR spectra of Hthnaphen, Hdhnaphen und Hnaphen in $CDCl_3$.
Figure S4. 600 MHz ^{1}H NMR spectrum of [Pt(naphen)Cl] in CD_2Cl_2.
Figure S5. 600 MHz ^{1}H NMR spectrum of [Pt(thnaphen)Cl] in CD_2Cl_2.
Figure S6. 600 MHz ^{1}H/^{1}H-COSY NMR spectrum of [Pt(thnaphen)Cl] in CD_2Cl_2.
Figure S7. 300 MHz ^{1}H NMR spectrum of [Pt(naphen)(C≡CPh)] in CD_2Cl_2.
Figure S8. 300 MHz ^{1}H NMR spectrum of [Pt(thnaphen)(C≡CPh)] in CD_2Cl_2.
Figure S9. Excerpt of the EI-MS (+) of [Pt(naphen)Cl] and simulation.
Figure S10. Excerpt from the EI-MS(+) (70 eV) of [Pt(thnaphen)Cl].
Figure S11. Simulated MS of [Pt(thnaphen)Cl]. $[Pt(naphen)]^+$, $[Pt(dhnaphen)]^+$, $[Pt(thnaphen)]^+$, and $[Pt(thnaphen)Cl]^+$.
Figure S12. Excerpt of the ESI-MS (+) of [Pt(thnaphen)Cl] in CH_3CN with simulation.
Figure S13. EI-MS (+) of [Pt(naphen)(C≡CPh)] with excerpt with simulation of $[M]^+$ at 575 m/z.
Figure S14. Excerpt of the EI-MS (+) of [Pt(thnaphen)(C≡CPh)] with simulation.
Figure S15. Cyclic voltammograms of Hthnaphen and Hnaphen in 0.1 M *n*-Bu_4NPF_6/THF solution.
Figure S16. Cyclic voltammograms of [Pt(thnaphen)Cl] in 0.1 M *n*-Bu_4NPF_6/THF and /CH_2Cl_2.
Figure S17. Cyclic voltammograms of [Pt(thnaphen)(C≡CPh)] in 0.1 M *n*-Bu_4NPF_6/THF and /CH_2Cl_2.
Figure S18. Cyclic voltammograms of [Pt(naphen)Cl] in 0.1 M *n*-Bu_4NPF_6/THF and /CH_2Cl_2.
Figure S19. Cyclic voltammograms of [Pt(bdq)Cl] in 0.1 M *n*-Bu_4NPF_6/THF and /CH_2Cl_2.
Figure S20. DFT-calculated contributions to the highest occupied molecular orbital (HOMO) and lowest unoccupied molecular orbital (LUMO) of [Pt(naphen)Cl] and [Pt(bdq)Cl] on B3LYP level.
Figure S21. UV-vis absorption spectra of Hthnaphen and Hnaphen in CH_2Cl_2.
Figure S22. UV-vis absorption spectra of [Pt(naphen)(C≡CPh)] in CH_2Cl_2.
Figure S23. UV-vis absorption spectra of [Pt(thnaphen)(C≡CPh)] in CH_2Cl_2.

Figure S24. Normalised emission spectra of [Pt(bdq)Cl] in a CH_2Cl_2 solution at RT and in a frozen glassy matrix of CH_2Cl_2:MeOH 1:1 at 77 K. λ_{ex} = 350 nm.
Figure S25. Normalised emission spectra of [Pt(naphen)Cl] in a CH_2Cl_2 solution at RT and in a frozen glassy matrix of CH_2Cl_2:MeOH 1:1 at 77 K. λ_{ex} = 350 nm.
Figure S26. Normalised emission spectra of [Pt(naphen)C≡CPh] in a CH_2Cl_2 solution at RT and in a frozen glassy matrix of CH_2Cl_2:MeOH 1:1 at 77 K. λ_{ex} = 350 nm.
Figure S27. Normalised emission spectra of [Pt(thnaphen)Cl] in a CH_2Cl_2 solution at RT and in a frozen glassy matrix of CH_2Cl_2:MeOH 1:1 at 77 K. λ_{ex} = 350 nm.
Figure S28. Normalised emission spectra of [Pt(thnaphen)C≡CPh] in a CH_2Cl_2 solution at RT and in a frozen glassy matrix of CH_2Cl_2:MeOH 1:1 at 77 K. λ_{ex} = 350 nm.
Figure S29. Normalised emission spectra of [Pt(bdq)Cl], [Pt(naphen)Cl], and [Pt(dba)(DMSO)] in CH_2Cl_2 (298 K) (λ_{ex} = 350 nm).
Figure S30. Time-resolved photoluminescence decay of [Pt(bdq)Cl] in an air-equilibrated CH_2Cl_2 solution at 298 K (c = 10^{-5} M), including the residuals (λ_{ex} = 376 nm, λ_{em} = 590 nm).
Figure S31. Time-resolved photoluminescence decay of [Pt(bdq)Cl] in an Ar-purged CH_2Cl_2 solution at 298 K (c = 10^{-5} M), including the residuals (λ_{ex} = 376 nm, λ_{em} = 590 nm).
Figure S32. Time-resolved photoluminescence decay of [Pt(bdq)Cl] in a frozen CH_2Cl_2:MeOH 1:1 glassy matrix at 77 (c = 10^{-5} M), including the residuals (λ_{ex} = 376 nm, λ_{em} = 590 nm).
Figure S33. Time-resolved photoluminescence decay of [Pt(naphen)Cl] in an air-equilibrated CH_2Cl_2 solution at 298 K (c = 10^{-5} M), including the residuals (λ_{ex} = 376 nm, λ_{em} = 640 nm).
Figure S34. Time-resolved photoluminescence decay of [Pt(naphen)Cl] in an Ar-purged CH_2Cl_2 solution at 298 K (c = 10^{-5} M), including the residuals (λ_{ex} = 376 nm, λ_{em} = 640 nm).
Figure S35. Time-resolved photoluminescence decay of [Pt(naphen)Cl] in a frozen CH_2Cl_2:MeOH 1:1 glassy matrix at 77 (c = 10^{-5} M), including the residuals (λ_{ex} = 376 nm, λ_{em} = 620 nm).
Figure S36. Time-resolved photoluminescence decay of [Pt(naphen)C≡CPh] in an air-equilibrated CH_2Cl_2 solution at 298 K (c = 10^{-5} M), including the residuals (λ_{ex} = 376 nm, λ_{em} = 650 nm).
Figure S37. Time-resolved photoluminescence decay of [Pt(naphen)C≡CPh] in an Ar-purged CH_2Cl_2 solution at 298 K (c = 10^{-5} M), including the residuals (λ_{ex} = 376 nm, λ_{em} = 650 nm).
Figure S38. Time-resolved photoluminescence decay of [Pt(naphen)C≡CPh] in a frozen CH_2Cl_2:MeOH 1:1 glassy matrix at 77 (c = 10^{-5} M), including the residuals (λ_{ex} = 376 nm, λ_{em} = 610 nm).
Figure S39. Time-resolved photoluminescence decay of [Pt(thnaphen)Cl] in an air-equilibrated CH_2Cl_2 solution at 298 K (c = 10^{-5} M), including the residuals (λ_{ex} = 376 nm, λ_{em} = 590 nm).
Figure S40. Time-resolved photoluminescence decay of [Pt(naphen)C≡CPh] in an Ar-purged CH_2Cl_2 solution at 298 K (c = 10^{-5} M), including the residuals (λ_{ex} = 376 nm, λ_{em} = 590 nm).
Figure S41. Time-resolved photoluminescence decay of [Pt(thnaphen)Cl] in a frozen CH_2Cl_2:MeOH 1:1 glassy matrix at 77 (c = 10^{-5} M), including the residuals (λ_{ex} = 376 nm, λ_{em} = 540 nm).
Figure S42. Time-resolved photoluminescence decay of [Pt(thnaphen)C≡CPh] in an air-equilibrated CH_2Cl_2 solution at 298 K (c = 10^{-5} M), including the residuals (λ_{ex} = 376 nm, λ_{em} = 600 nm).
Figure S43. Time-resolved photoluminescence decay of [Pt(naphen)C≡CPh] in an Ar-purged CH_2Cl_2 solution at 298 K (c = 10^{-5} M), including the residuals (λ_{ex} = 376 nm, λ_{em} = 600 nm).
Figure S44. Time-resolved photoluminescence decay of [Pt(thnaphen)C≡Ph] in a frozen CH_2Cl_2:MeOH 1:1 glassy matrix at 77 (c = 10^{-5} M), including the residuals (λ_{ex} = 376 nm, λ_{em} = 550 nm).
Figure S45. Left: Nanosecond time-resolved emission spectra superimposed to the inverted steady-state spectrum in black (A) and transient absorption spectra (B) of [Pt(naphen)Cl] in CH_2Cl_2 at selected time points. Black/grey line refers to superimposed inverted steady state emission/absorption spectra respectively. Right: Decay fits at selected wavelengths. Please note: The figure presents data, which were recorded in the presence of trace amount of ligand. The residual free ligand in the sample leads to the short-lived emission band centered at 410 nm.

Supporting Tables

Table S1. UV-vis absorptions of the ligands and [Pt(L)X] complexes.

Supporting Figures

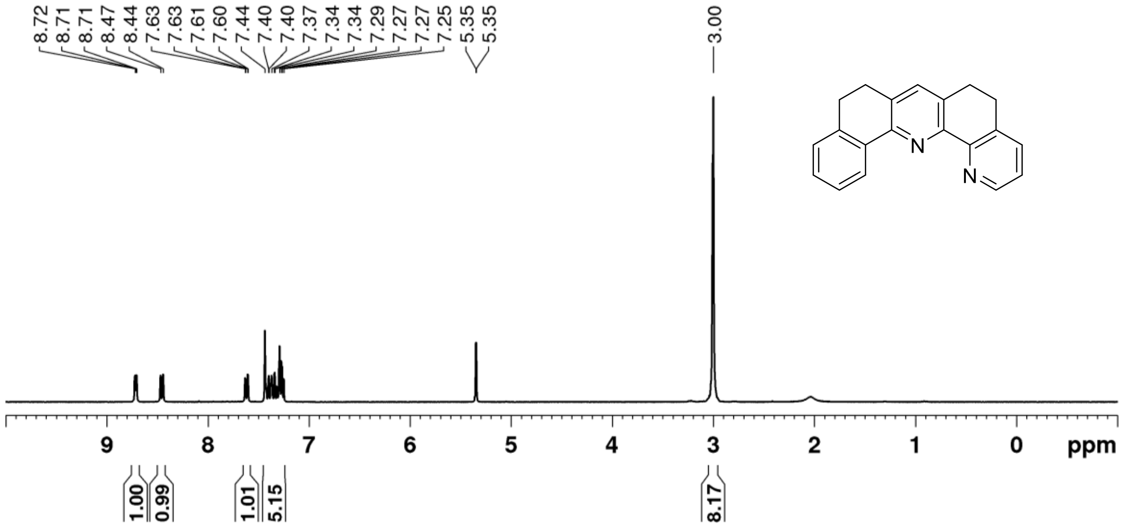

Figure S1. 300 MHz ^{1}H NMR spectrum of 5,6,8,9-tetrahydro-naphtho[1,2-*b*][1,10]phenanthroline (Hthnaphen) in CD_2Cl_2.

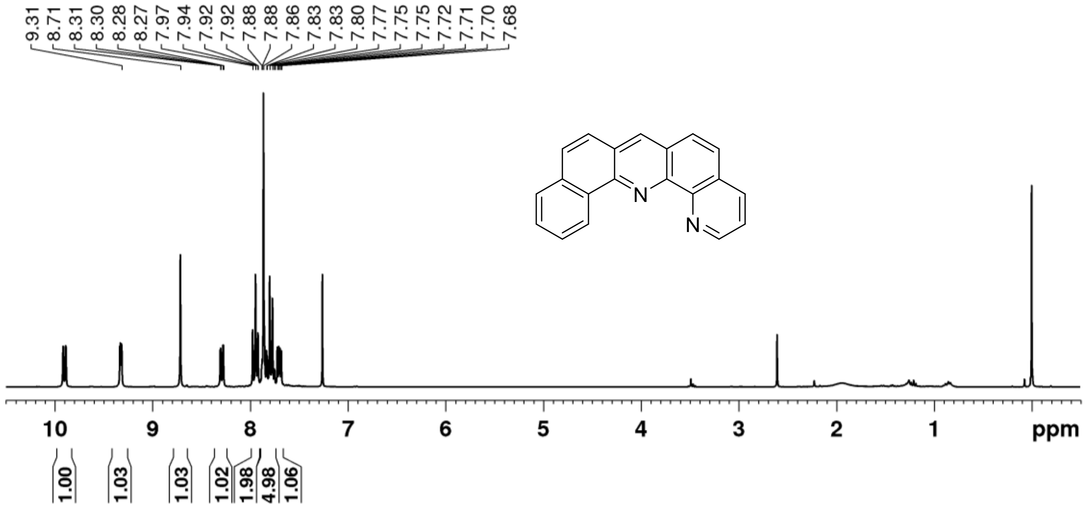

Figure S2. 300 MHz ^{1}H NMR spectrum of naphtho[1,2-*b*][1,10]phenanthroline (Hnaphen) in $CDCl_3$.

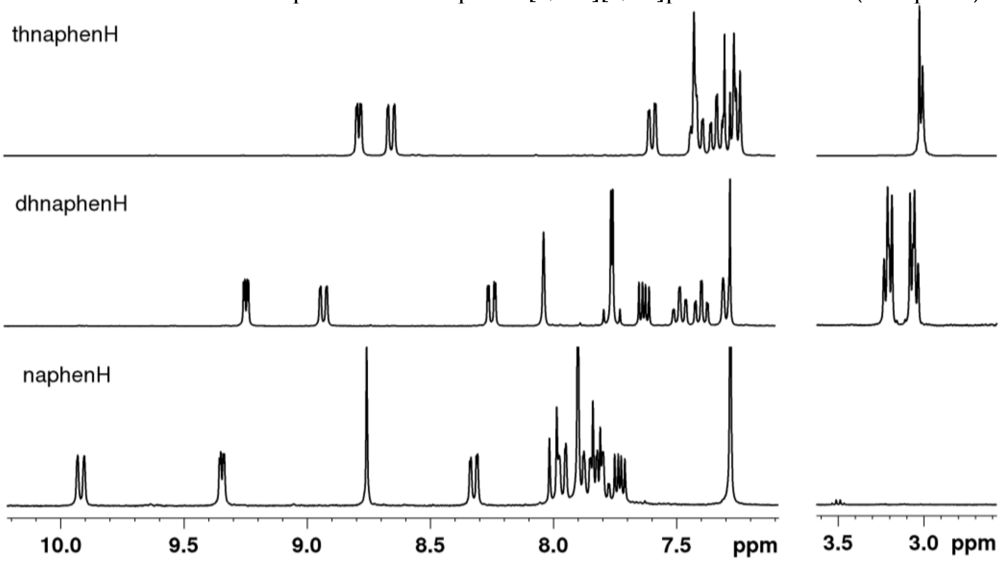

Figure S3. 300 MHz ^{1}H NMR spectra of Hthnaphen, Hdhnaphen und Hnaphen in $CDCl_3$.

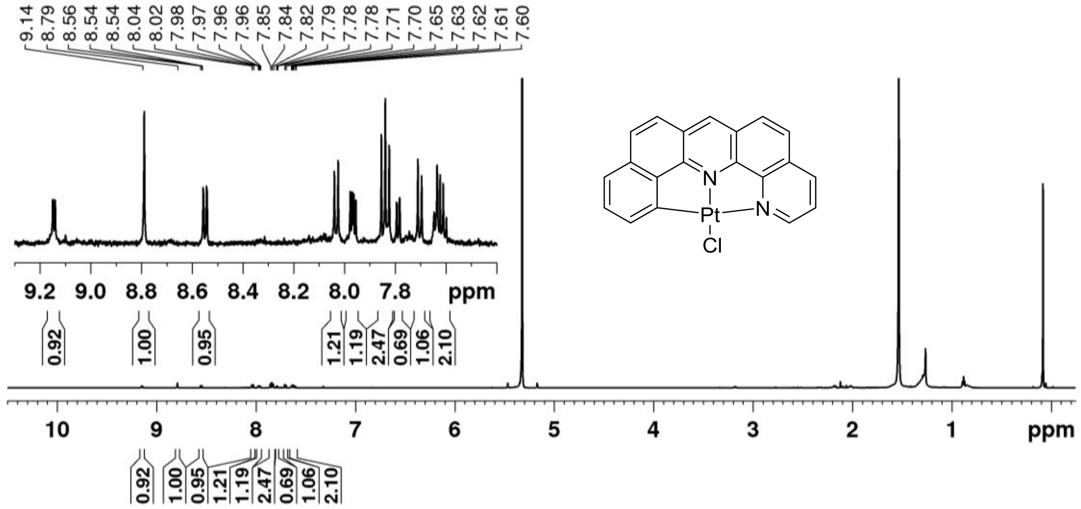

Figure S4. 600 MHz ^{1}H NMR spectrum of [Pt(naphen)Cl] in CD_2Cl_2.

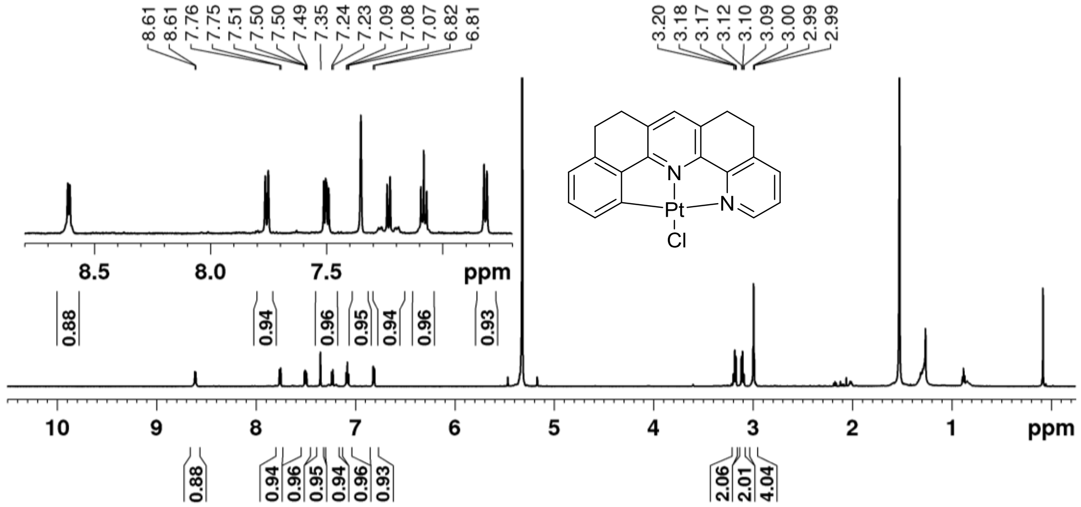

Figure S5. 600 MHz ^{1}H NMR spectrum of [Pt(thnaphen)Cl] in CD_2Cl_2.

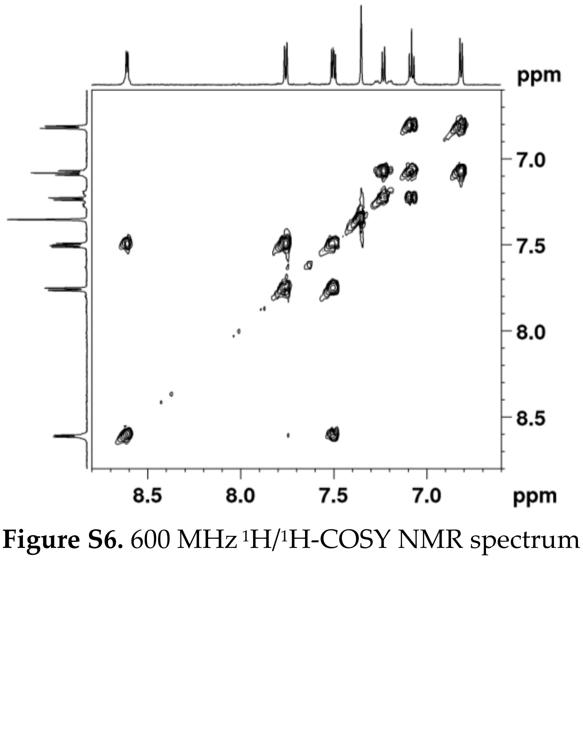

Figure S6. 600 MHz ^{1}H/^{1}H-COSY NMR spectrum of [Pt(thnaphen)Cl] in CD_2Cl_2.

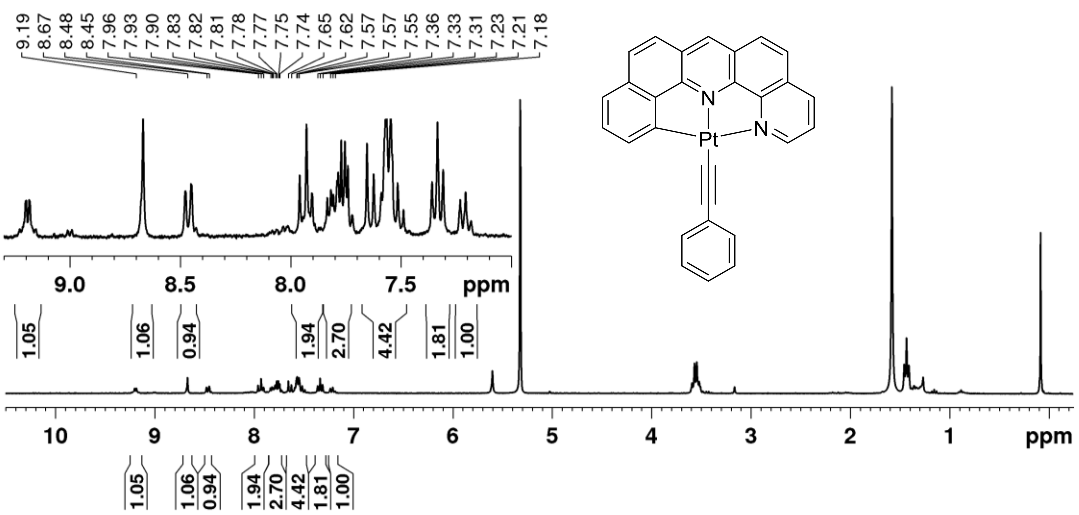

Figure S7. 300 MHz ^{1}H NMR spectrum of [Pt(naphen)(C≡CPh)] in CD_2Cl_2.

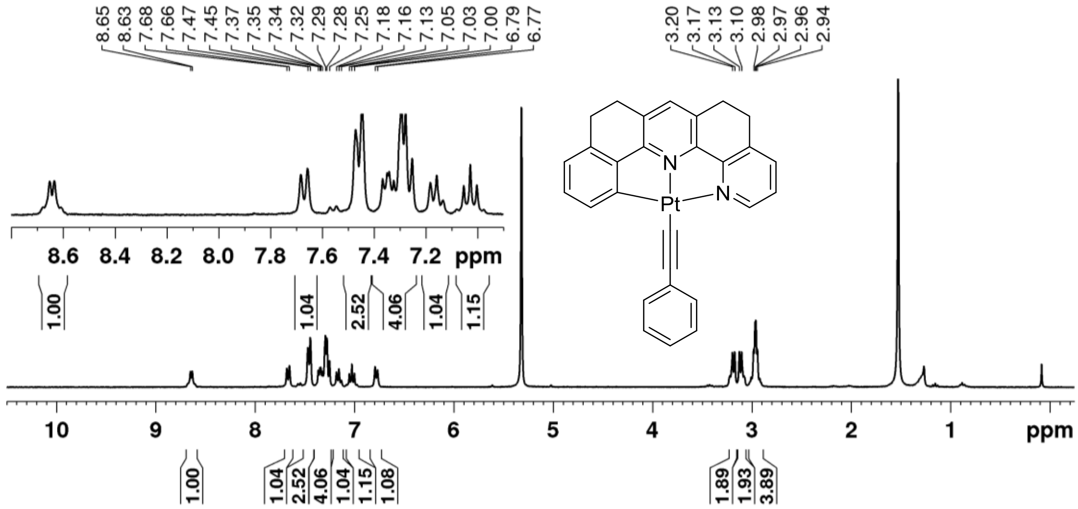

Figure S8. 300 MHz ^{1}H NMR spectrum of [Pt(thnaphen)(C≡CPh)] in CD_2Cl_2.

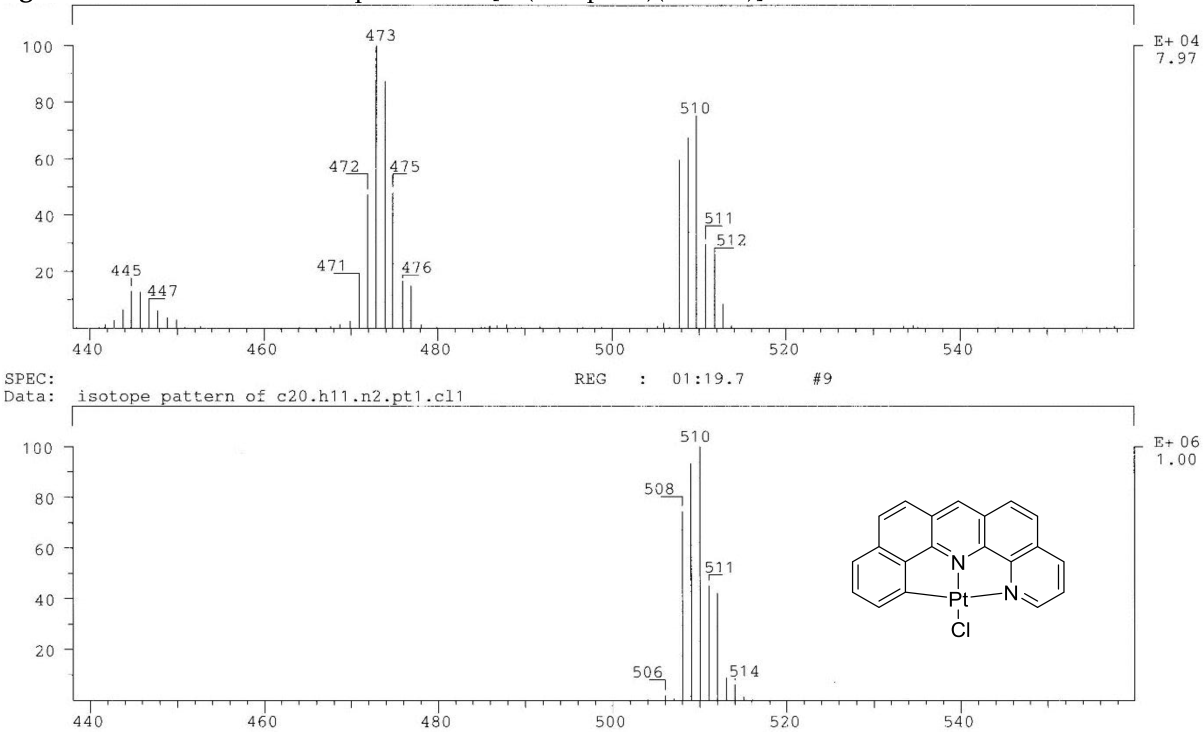

Figure S9. Excerpt of the EI-MS (+) of [Pt(naphen)Cl] (top) with simulation (bottom).

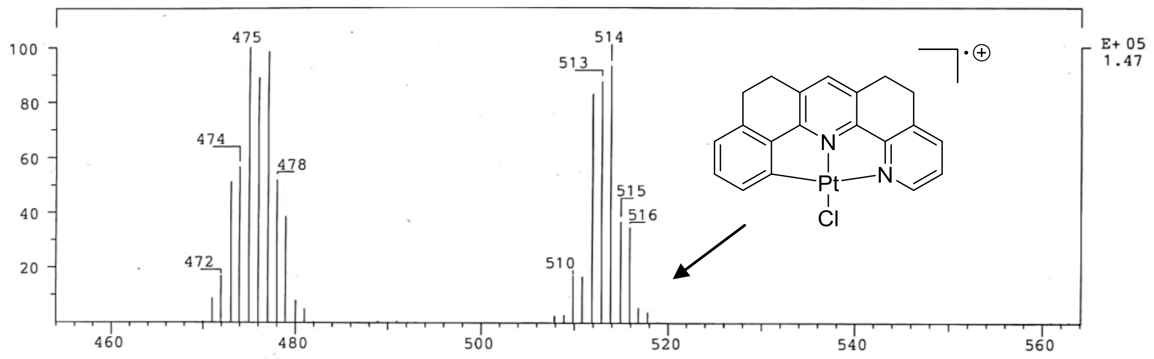

Figure S10. Excerpt from the EI-MS(+) (70 eV) of [Pt(thnaphen)Cl].

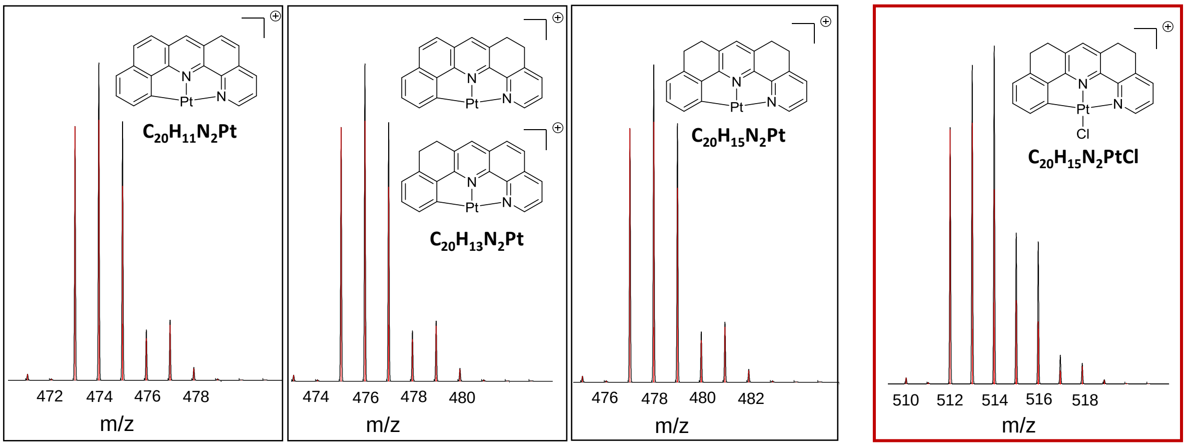

Figure S11. Simulated MS of [Pt(thnaphen)Cl]. [Pt(naphen)]$^+$, [Pt(dhnaphen)]$^+$, [Pt(thnaphen)]$^+$, and [Pt(thnaphen)Cl]$^+$.

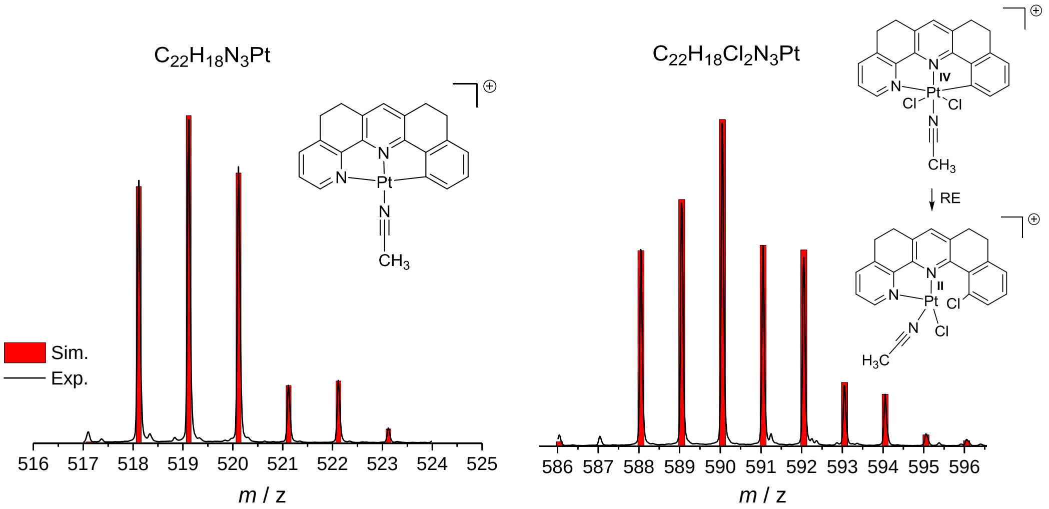

Figure S12. Excerpt of the ESI-MS (+) of [Pt(thnaphen)Cl] in CH3CN with simulation (in red).

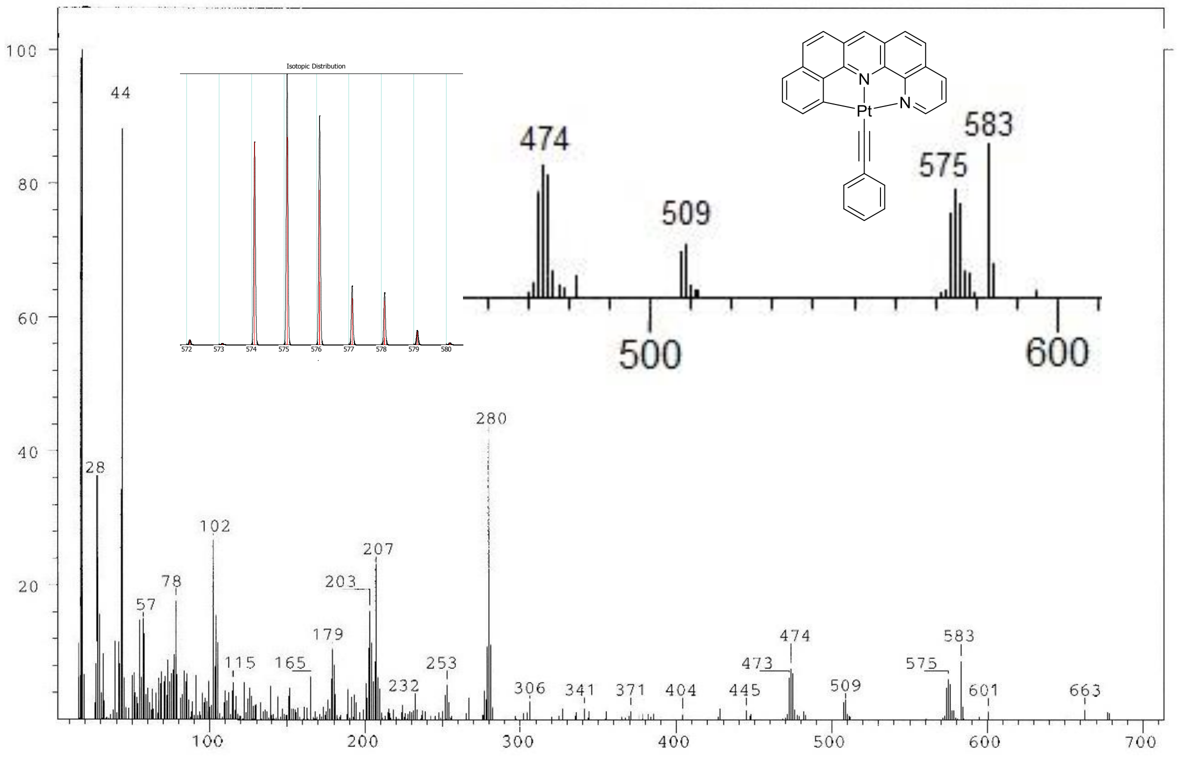

Figure S13. EI-MS (+) of [Pt(naphen)(C≡CPh)] with excerpt with simulation of $[M]^+$ at 575 m/z.

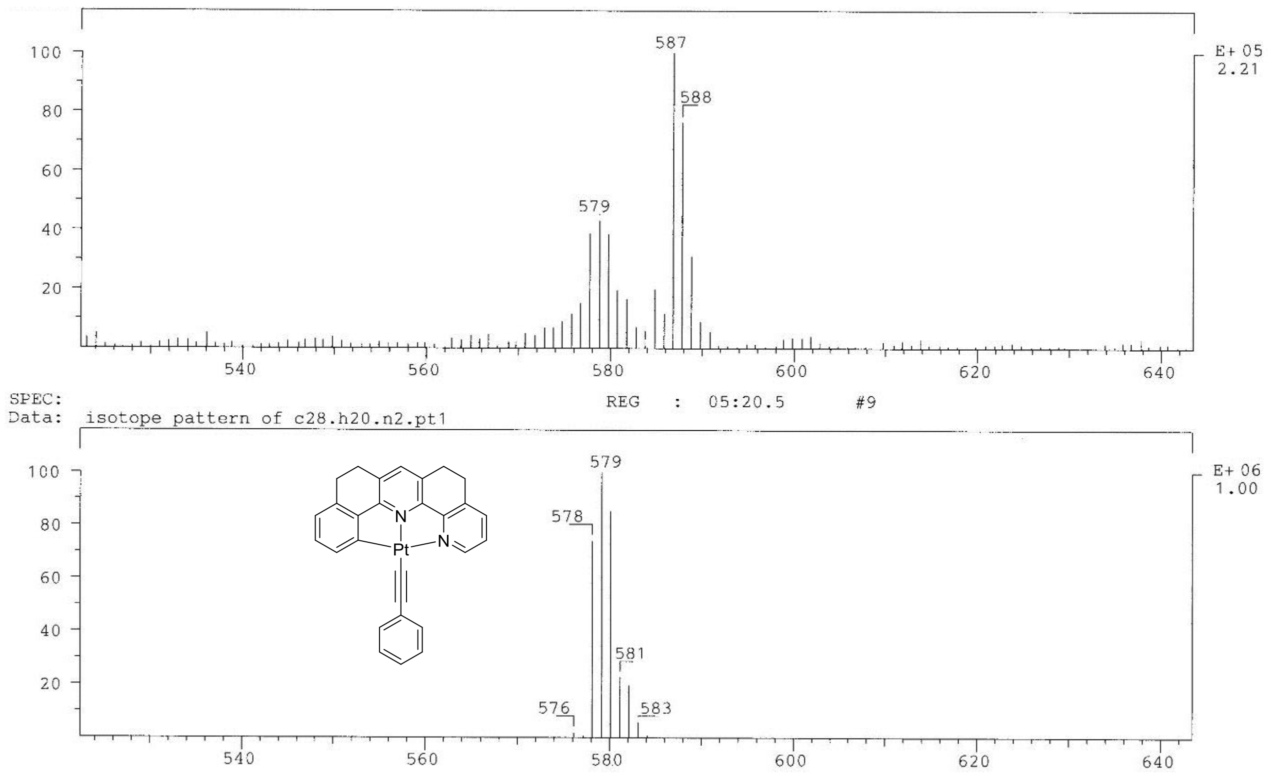

Figure S14. Excerpt of the EI-MS (+) of [Pt(thnaphen)(C≡CPh)] (top) with simulation (bottom).

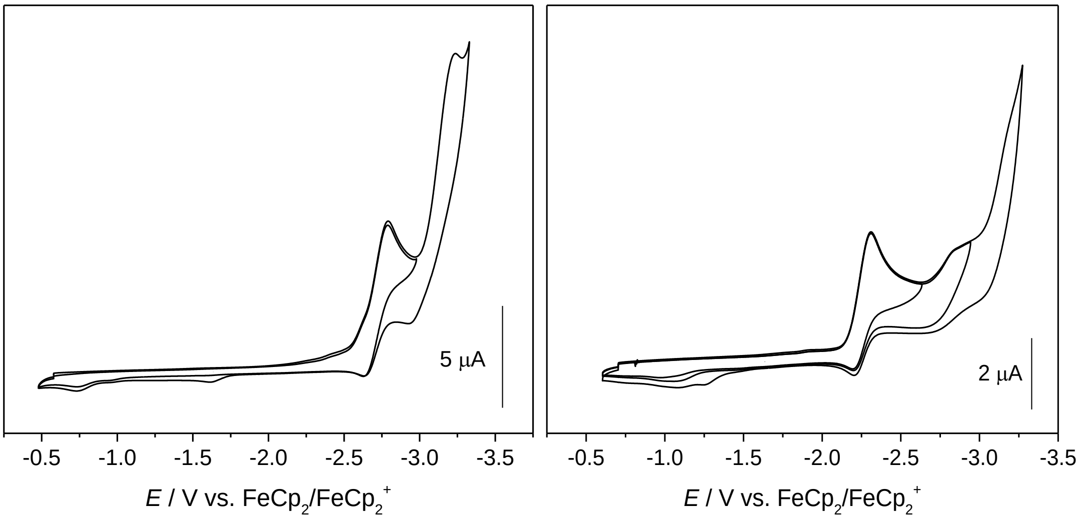

Figure S15. Cyclic voltammograms of Hthnaphen (left) and Hnaphen (right) in 0.1 M *n*-Bu_4NPF_6/THF solution.

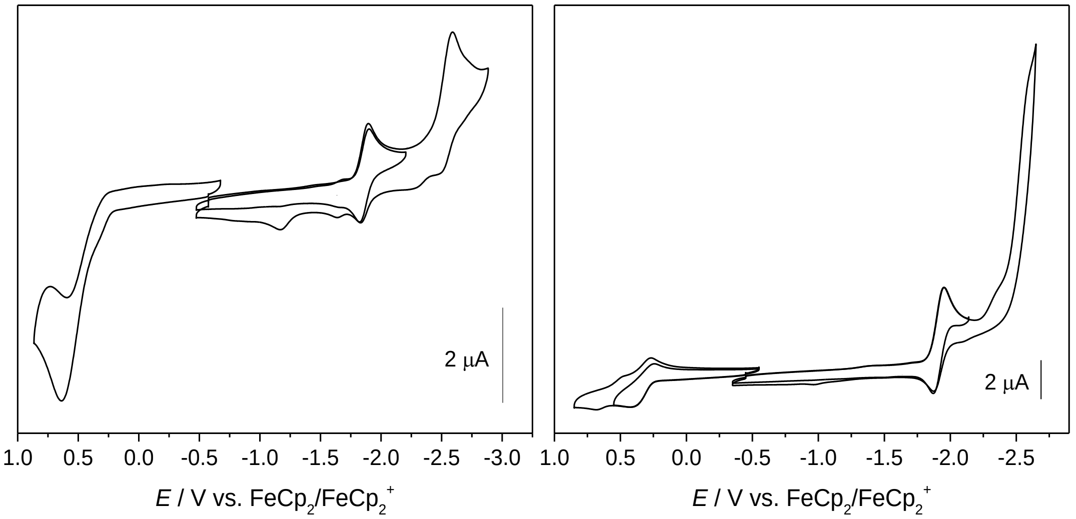

Figure S16. Cyclic voltammograms of [Pt(thnaphen)Cl] in 0.1 M *n*-Bu_4NPF_6/THF (left) and in 0.1 M *n*-Bu_4NPF_6/CH_2Cl_2 (right).

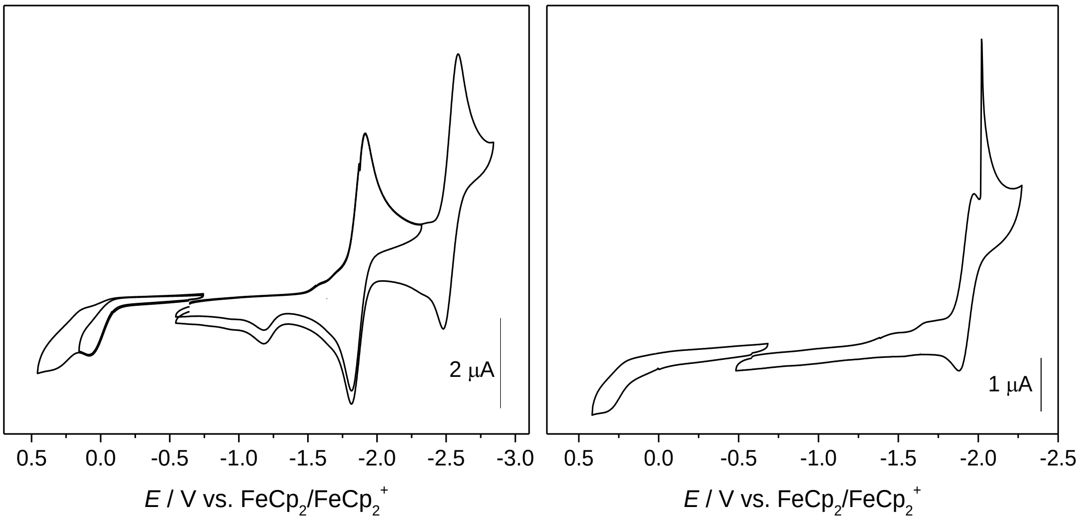

Figure S17. Cyclic voltammograms of [Pt(thnaphen)(C≡CPh)] in 0.1 M *n*-Bu_4NPF_6/THF (left) and in 0.1 M *n*-Bu_4NPF_6/CH_2Cl_2 (right).

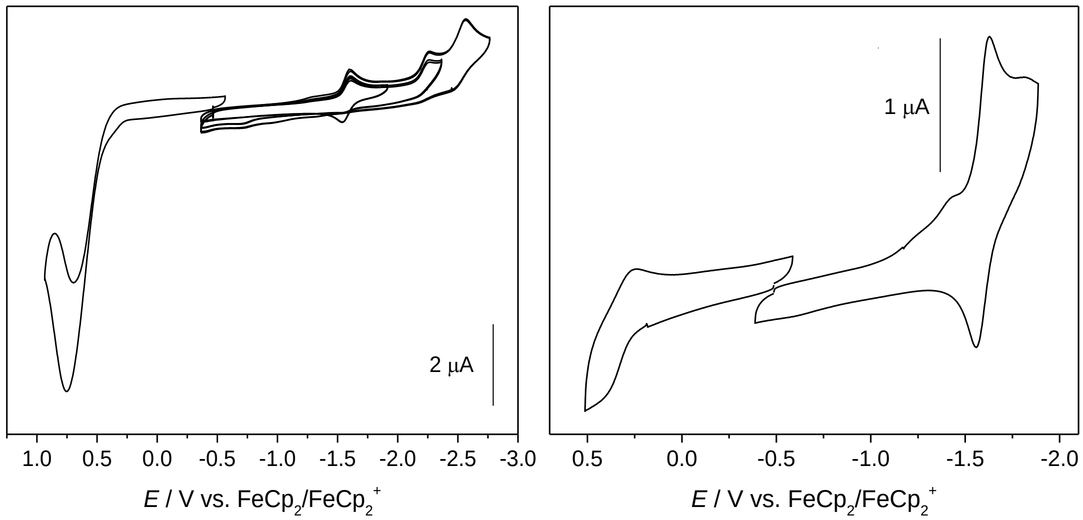

Figure S18. Cyclic voltammograms of [Pt(naphen)Cl] in 0.1 M *n*-Bu_4NPF_6/THF (left) and in 0.1 M *n*-Bu_4NPF_6/CH_2Cl_2 (right).

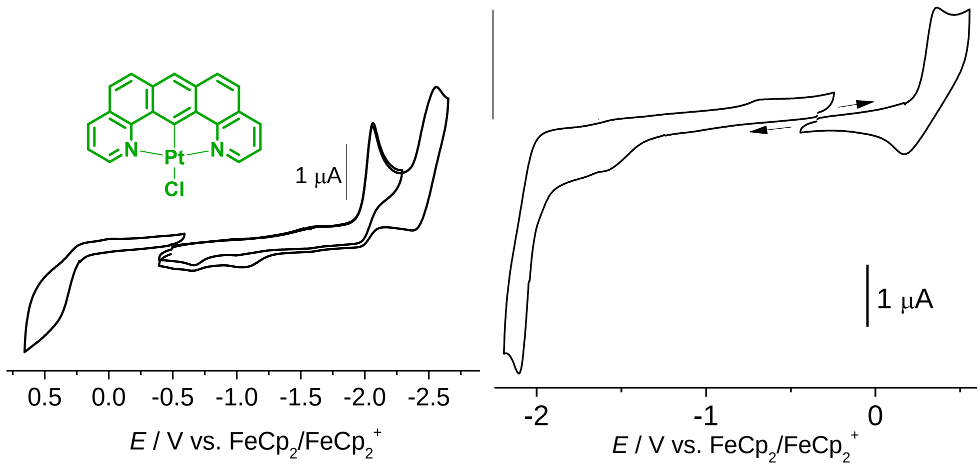

Figure S19. Cyclic voltammograms of [Pt(bdq)Cl] in 0.1 M *n*-Bu_4NPF_6/THF (left) and /CH_2Cl_2 (right).

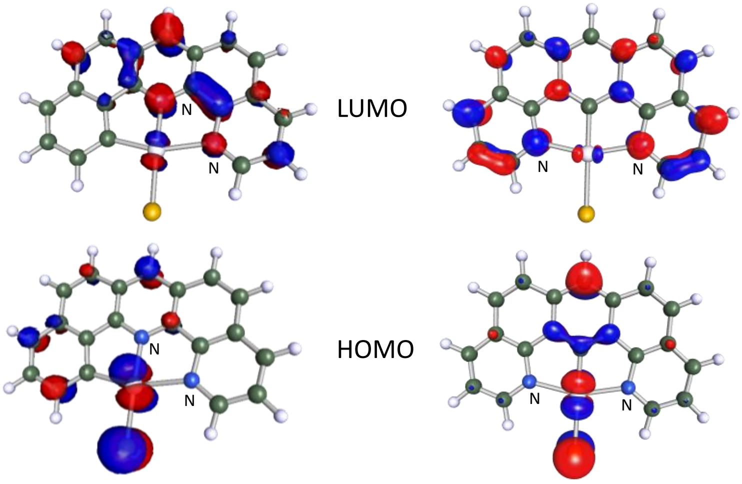

Figure S20. DFT-calculated contributions to the highest occupied molecular orbital (HOMO) and lowest unoccupied molecular orbital (LUMO) of [Pt(naphen)Cl] (left) and [Pt(bdq)Cl] (right) on B3LYP level using the basis sets def2-TZV(P) for C, H, N, S and LAN-L2DZ für Pt (ECP: Hay/Wadt (n=1)). Isosurface: 0.05.

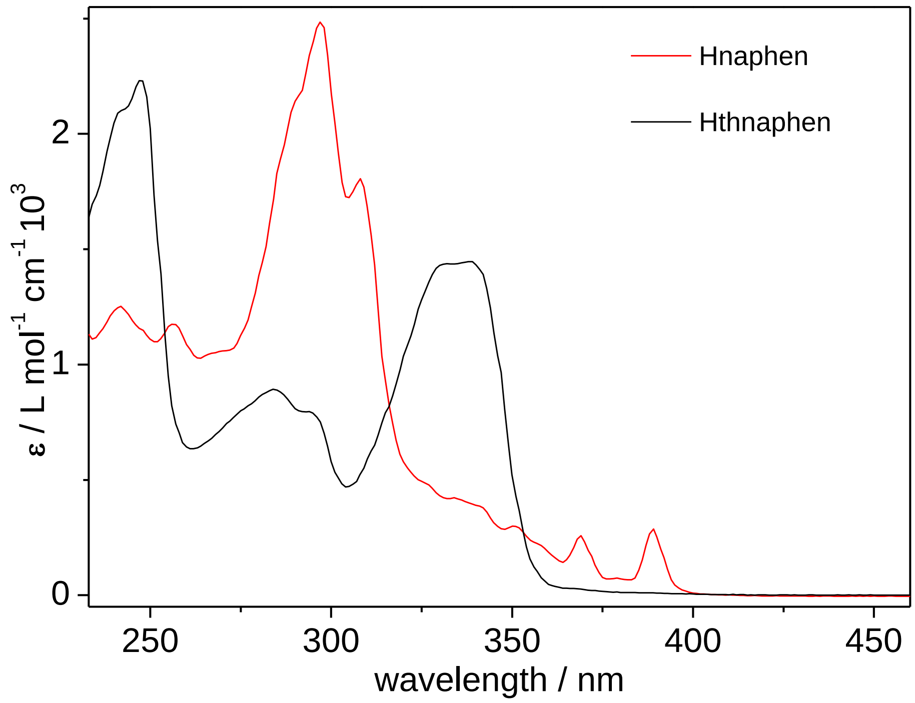

Figure S21. UV-vis absorption spectra of Hthnaphen (black) and Hnaphen (red) in CH_2Cl_2 (298 K).

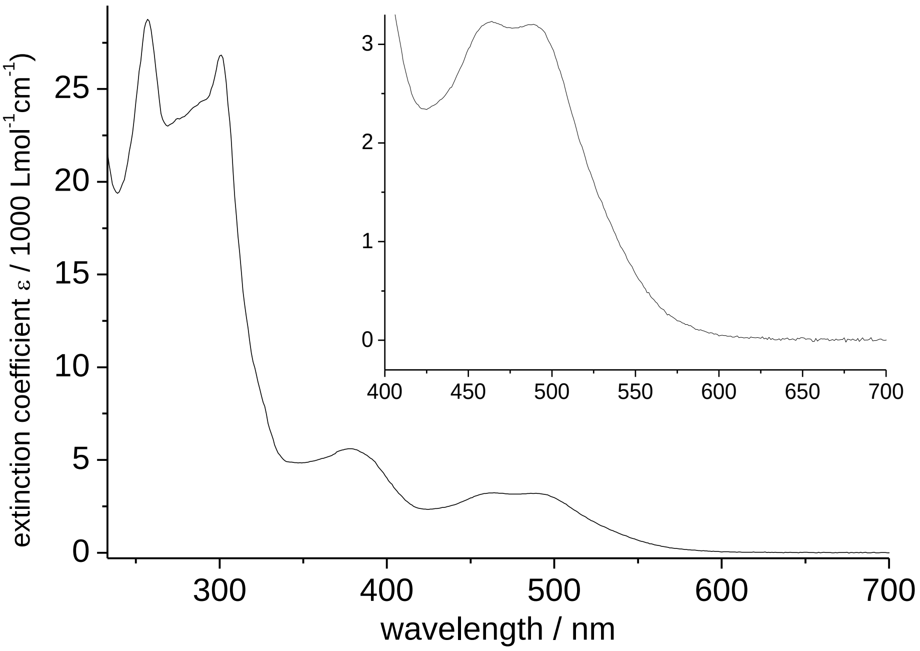

Figure S22. UV-vis absorption spectra of [Pt(naphen)(C≡CPh)] in CH_2Cl_2 (298 K). Y-Achse: Molar absorption coefficient (oder nur epsilon, wie Fig. S21)

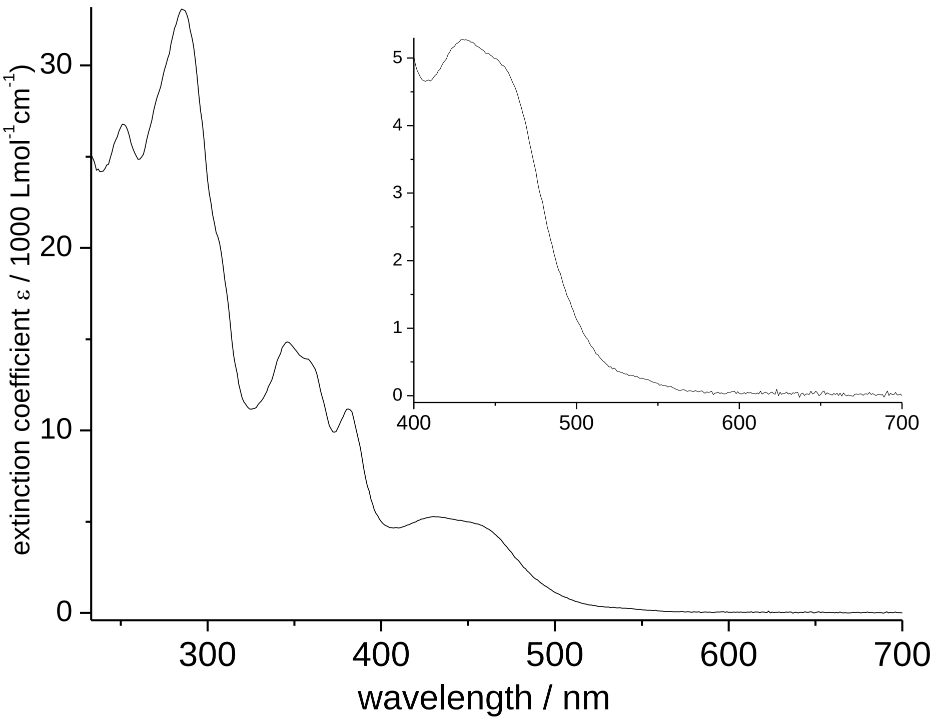

Figure S23. UV-vis absorption spectra of [Pt(thnaphen)(C≡CPh)] in CH_2Cl_2 (298 K). Y-Achse: wie Fig. S21

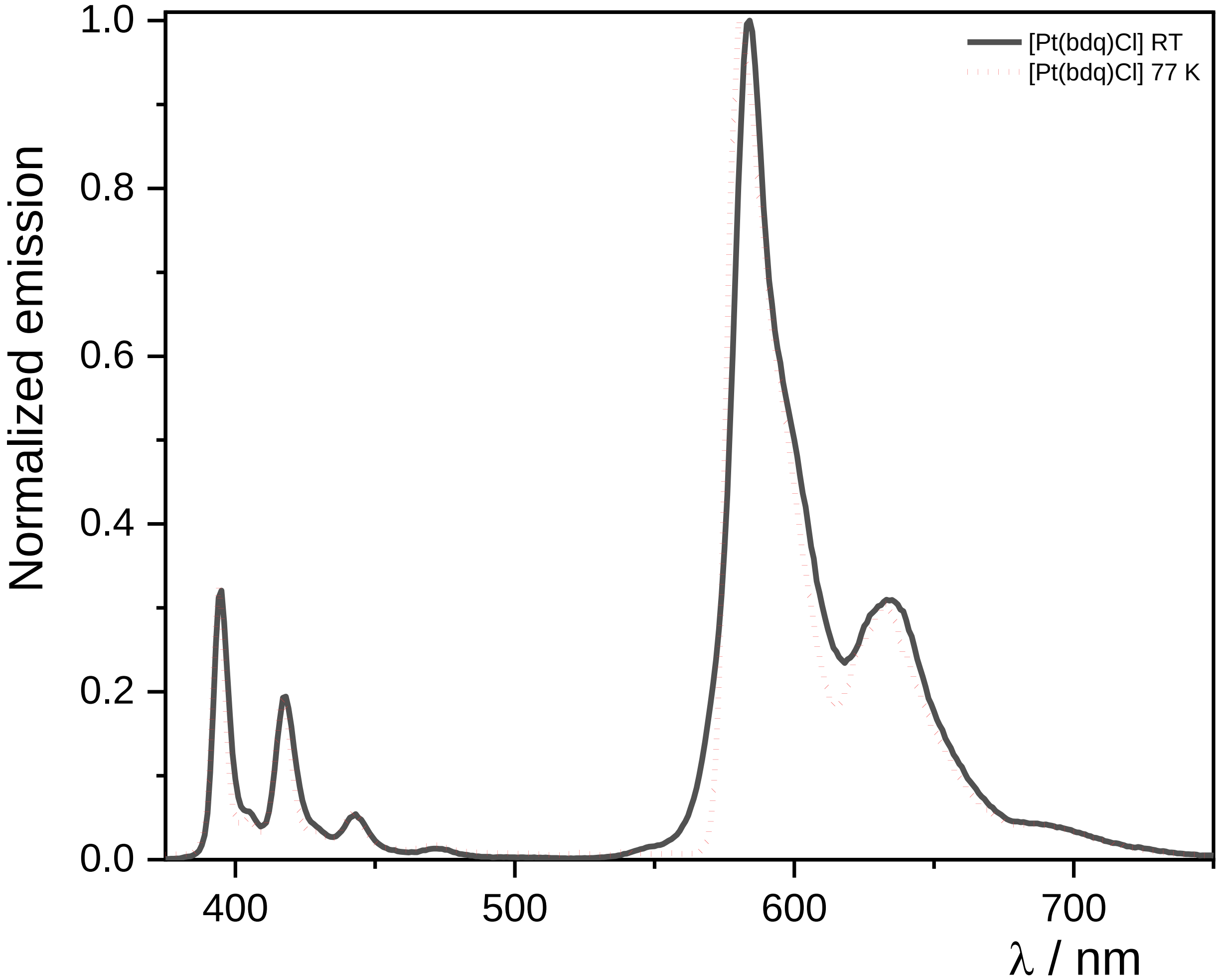

Figure S24. Normalised emission spectra of [Pt(bdq)Cl] in a CH_2Cl_2 solution at RT (black solid line) and in a frozen glassy matrix of CH_2Cl_2:MeOH 1:1 at 77 K (red dotted line). λ_{ex} = 350 nm. The emission at around 400 nm corresponds to the ligand Hbdq.

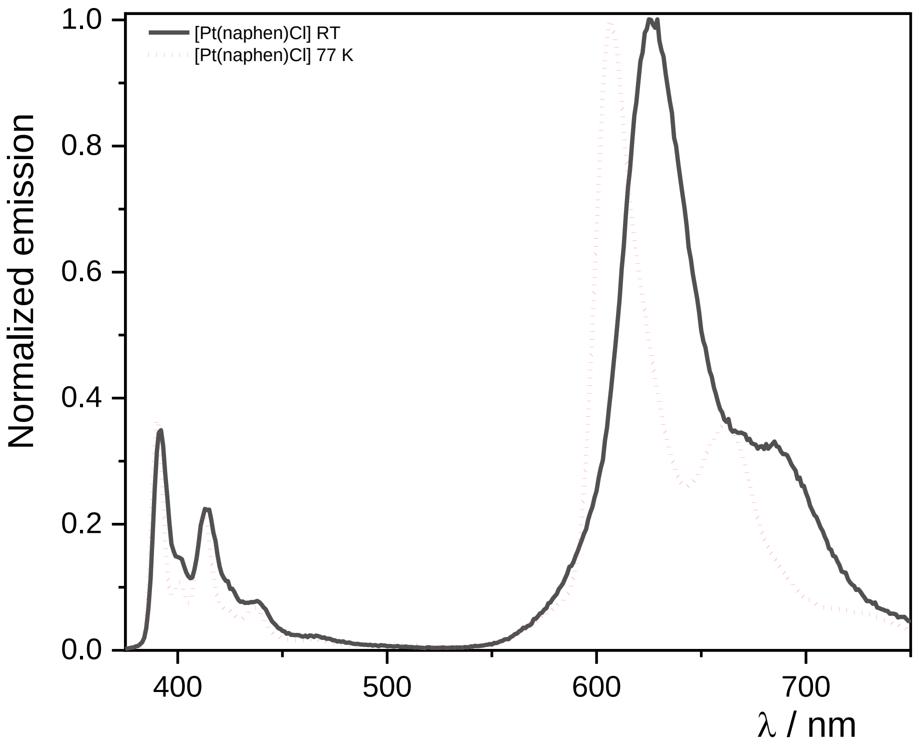

Figure S25. Normalised emission spectra of [Pt(naphen)Cl] in a CH_2Cl_2 solution at RT (black solid line) and in a frozen glassy matrix of CH_2Cl_2:MeOH 1:1 at 77 K (red dotted line). λ_{ex} = 350 nm. The emission at around 400 nm corresponds to the ligand Hnaphen.

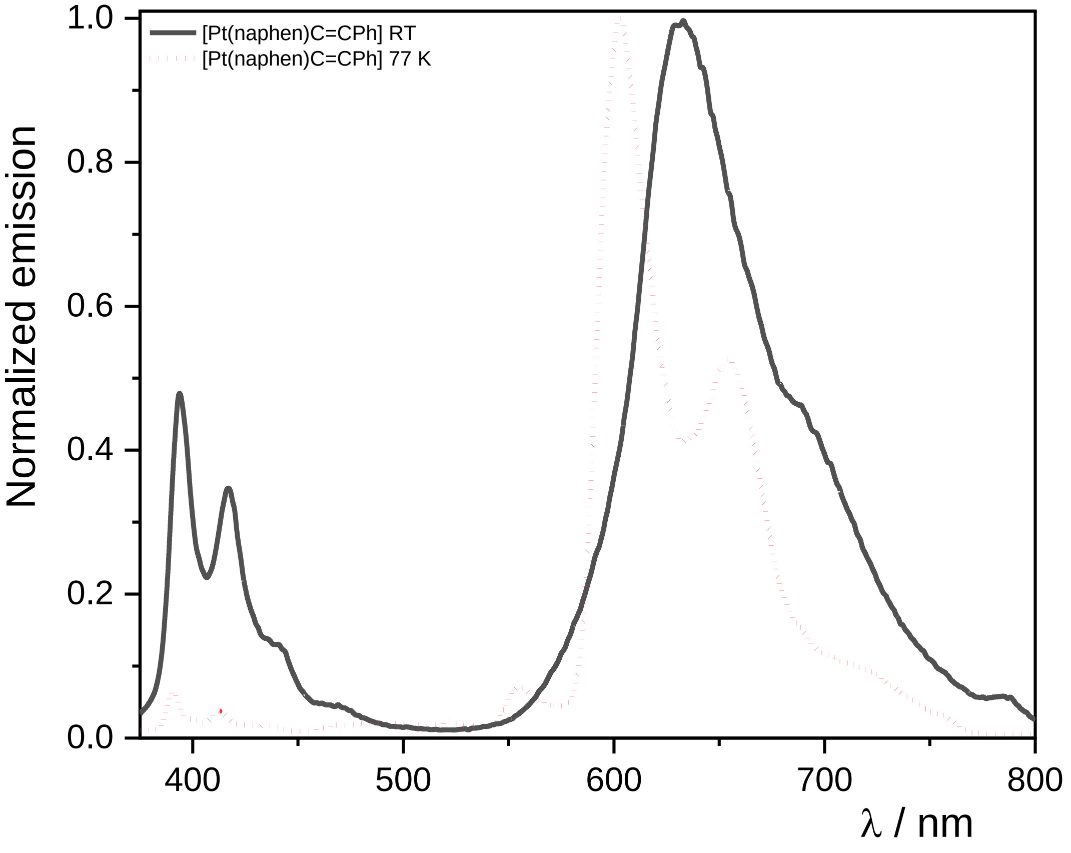

Figure S26. Normalised emission spectra of [Pt(naphen)C≡CPh] in a CH_2Cl_2 solution at RT (black solid line) and in a frozen glassy matrix of CH_2Cl_2:MeOH 1:1 at 77 K (red dotted line). λ_{ex} = 350 nm. The emission at around 400 nm corresponds to the ligand Hnaphen.

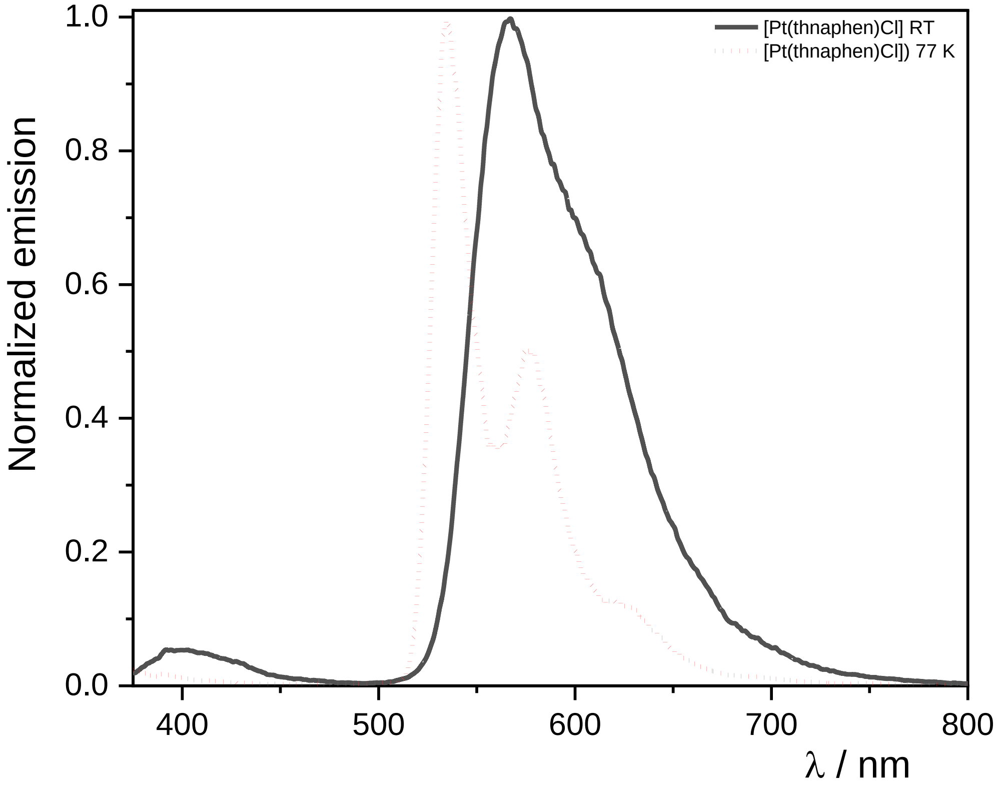

Figure S27. Normalised emission spectra of [Pt(thnaphen)Cl] in a CH_2Cl_2 solution at RT (black solid line) and in a frozen glassy matrix of CH_2Cl_2:MeOH 1:1 at 77 K (red dotted line). λ_{ex} = 350 nm. The emission at around 400 nm corresponds to the ligand Hthnaphen.

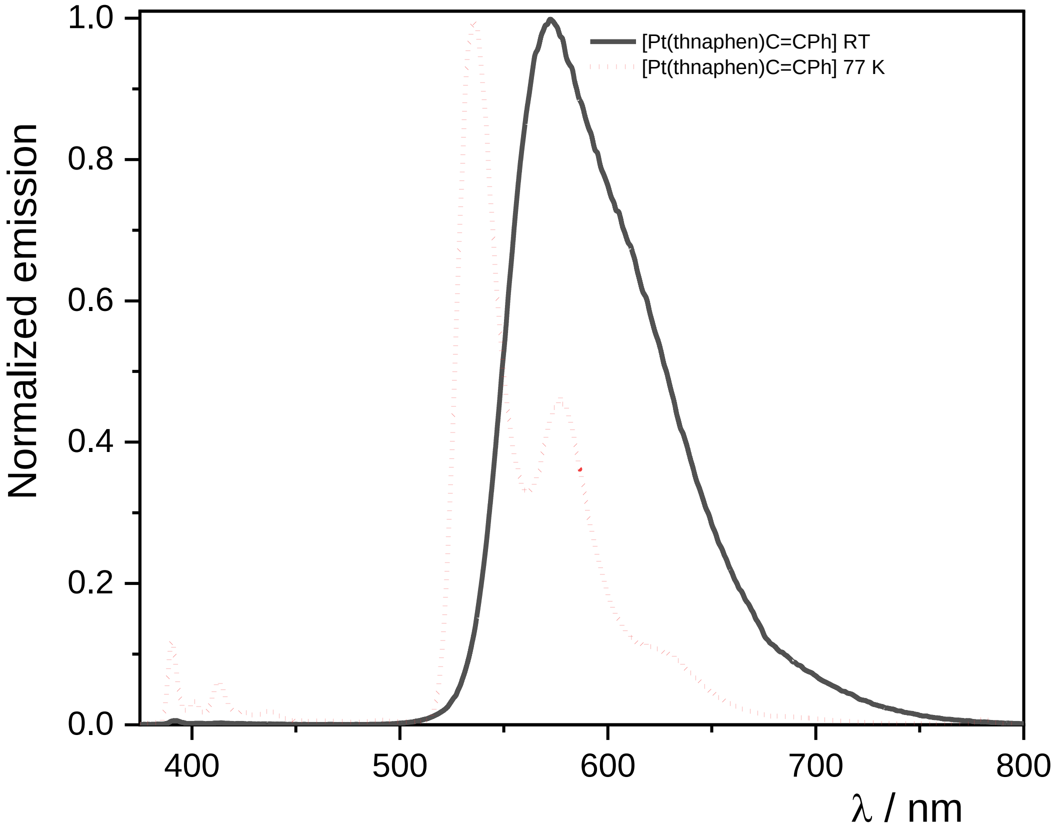

Figure S28. Normalised emission spectra of [Pt(thnaphen)C≡CPh] in a CH_2Cl_2 solution at RT (black solid line) and in a frozen glassy matrix of CH_2Cl_2:MeOH 1:1 at 77 K (red dotted line). λ_{ex} = 350 nm. The emission at around 400 nm corresponds to the ligand Hthnaphen.

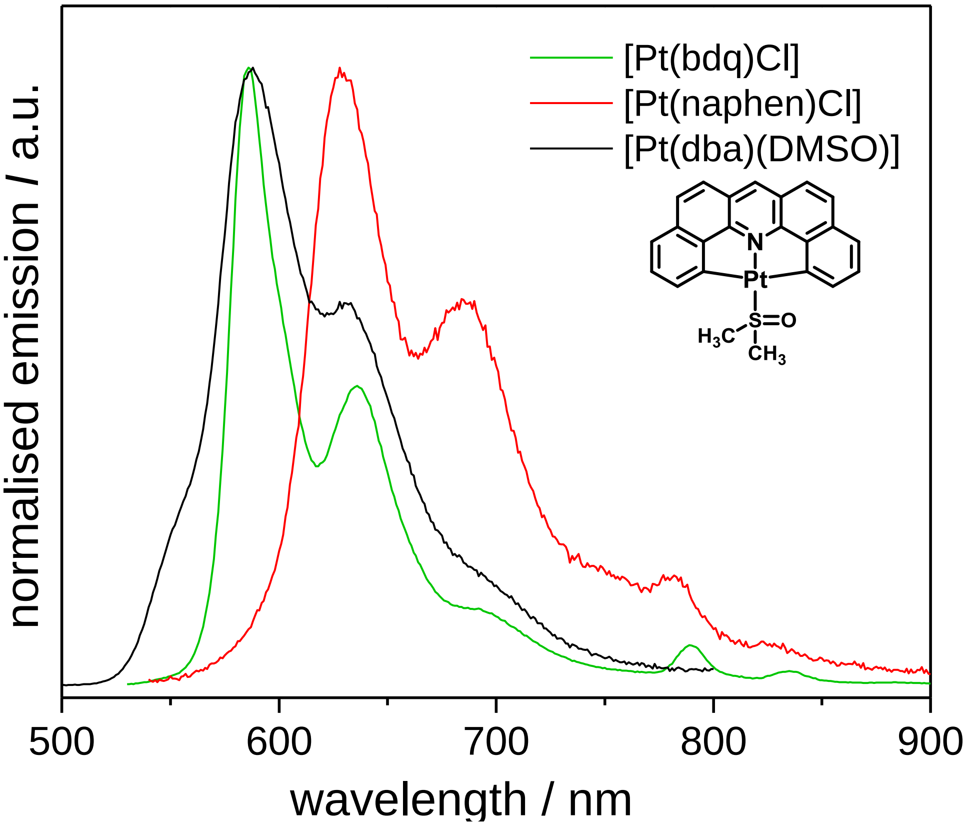

Figure S29. Normalised emission spectra of [Pt(bdq)Cl], [Pt(naphen)Cl], and [Pt(dba)(DMSO)][1] in CH_2Cl_2 (298 K) (λ_{exc} = 350 nm).

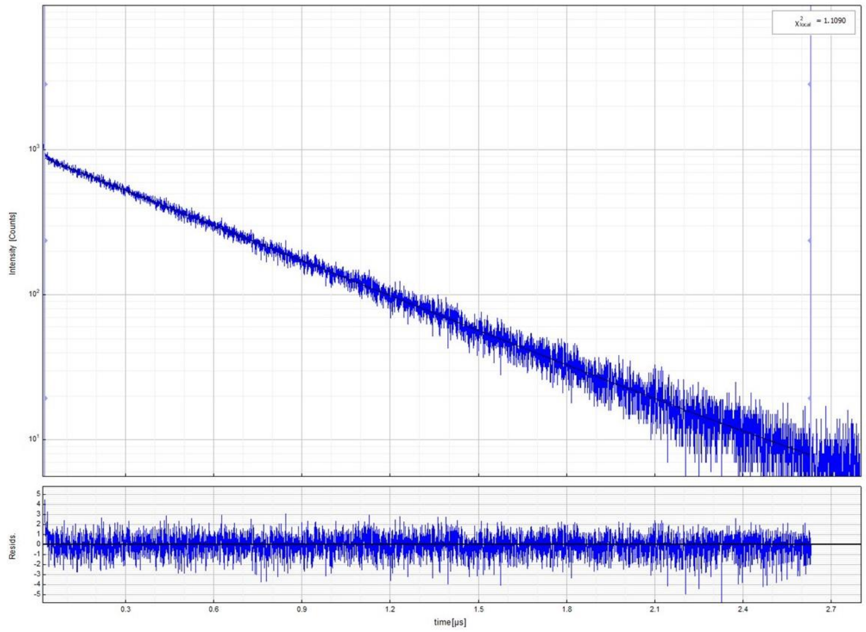

Parameter	Value	Error
A_1[kCnts/Chnl]	0.8798	± 0.0026
τ_1[ns]	533.9	± 2.5
I_1[kCnts]	917.5	± 2.2
$A_{Rel\,1}$[%]	100.0000	± 0.0000
$I_{Rel\,1}$[%]	100.0000	± 0.0000
$Bkgr_{Dec}$[kCnts]	0.0012	± 0.0002
τ_{AvInt}[ns]	533.9	± 2.5
τ_{AvAmp}[ns]	533.9	± 2.5

Figure S30. Left: Time-resolved photoluminescence decay of [Pt(bdq)Cl] in an air-equilibrated CH_2Cl_2 solution at 298 K (c = 10^{-5} M), including the residuals (λ_{ex} = 376 nm, λ_{em} = 590 nm). Right: Fitting parameters including pre-exponential factors and confidence limits.

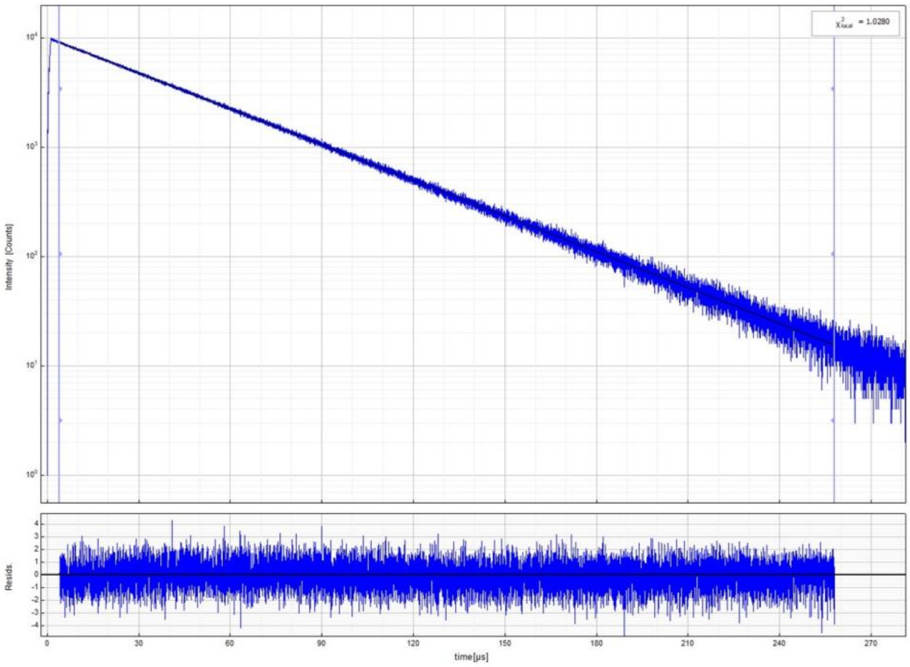

Parameter	Value	Error
A_1[kCnts/Chnl]	9.0783	± 0.0075
τ_1[ns]	39928	± 27
I_1[kCnts]	22654.7	± 8.0
$A_{Rel\,1}$[%]	100.0000	± 0.0000
$I_{Rel\,1}$[%]	100.0000	± 0.0000
$Bkgr_{Dec}$[kCnts]	-0.0002	± 0.0002
$\tau_{Av\,Int}$[ns]	39928	± 27
$\tau_{Av\,Amp}$[ns]	39928	± 27

Figure S31. Left: Time-resolved photoluminescence decay of [Pt(bdq)Cl] in an Ar-purged CH_2Cl_2 solution at 298 K (c = 10^{-5} M), including the residuals (λ_{ex} = 376 nm, λ_{em} = 590 nm). Right: Fitting parameters including pre-exponential factors and confidence limits.

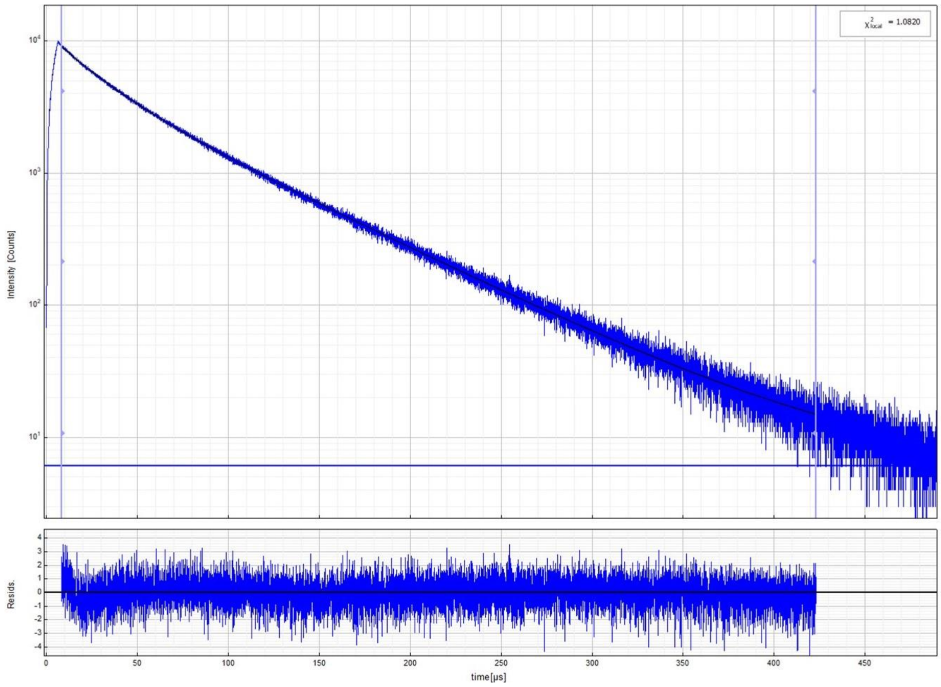

Parameter	Value	Error
A_1[kCnts/Chnl]	4.958	± 0.046
τ_1[ns]	65584	± 248
I_1[kCnts]	10162	± 56
$A_{Rel\,1}$[%]	54.87	± 0.46
$I_{Rel\,1}$[%]	77.12	± 0.49
A_2[kCnts/Chnl]	4.078	± 0.038
τ_2[ns]	23650	± 295
I_2[kCnts]	3014	± 66
$A_{Rel\,2}$[%]	45.13	± 0.46
$I_{Rel\,2}$[%]	22.88	± 0.49
$Bkgr_{Dec}$[kCnts]	0.0061	± 0.0003
$\tau_{Av\,Int}$[ns]	55992	± 57
$\tau_{Av\,Amp}$[ns]	46659	± 77

Figure S32. Left: Time-resolved photoluminescence decay of [Pt(bdq)Cl] in a frozen CH_2Cl_2:MeOH 1:1 glassy matrix at 77 (c = 10^{-5} M), including the residuals (λ_{ex} = 376 nm, λ_{em} = 590 nm). Right: Fitting parameters including pre-exponential factors and confidence limits.

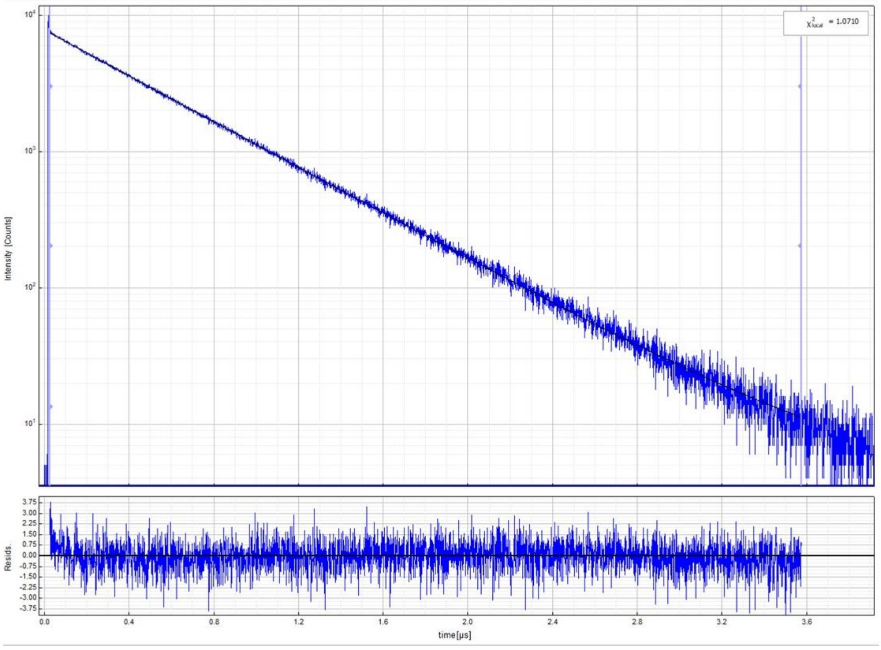

Parameter	Value	Error
A_1[kCnts/Chnl]	7.379	± 0.015
τ_1[ns]	516.90	± 0.95
I_1[kCnts]	3814.0	± 3.2
$A_{Rel\,1}$[%]	100.0000	± 0.0000
$I_{Rel\,1}$[%]	100.0000	± 0.0000
$Bkgr_{Dec}$[kCnts]	0.0035	± 0.0003
$\tau_{Av\,Int}$[ns]	516.90	± 0.95
$\tau_{Av\,Amp}$[ns]	516.90	± 0.95

Figure S33. Left: Time-resolved photoluminescence decay of [Pt(naphen)Cl] in an air-equilibrated CH_2Cl_2 solution at 298 K (c = 10^{-5} M), including the residuals (λ_{ex} = 376 nm, λ_{em} = 640 nm). Right: Fitting parameters including pre-exponential factors and confidence limits.

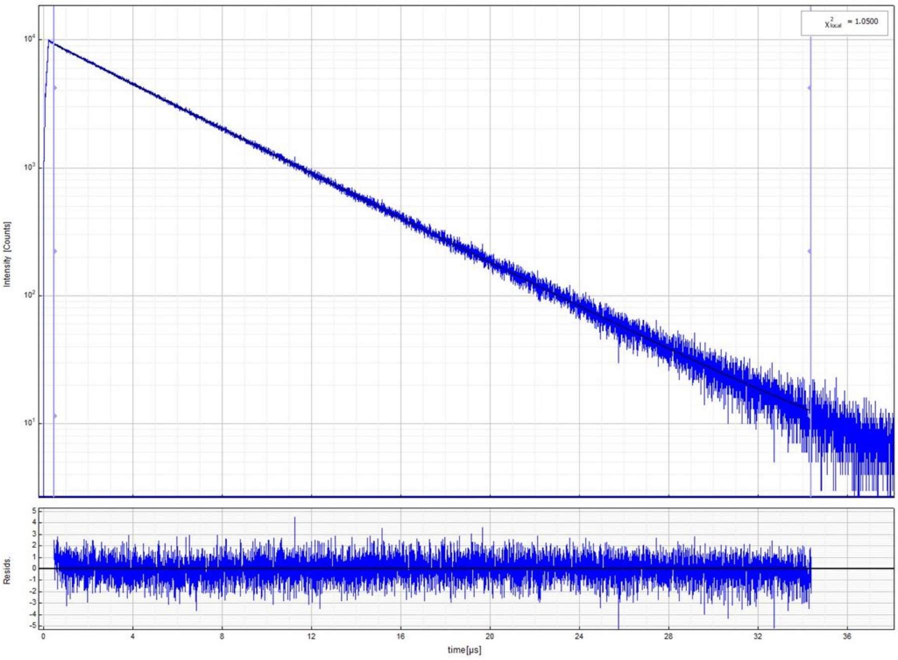

Parameter	Value	Error
A_1[kCnts/Chnl]	9.2214	± 0.0065
τ_1[ns]	4955.3	± 2.1
I_1[kCnts]	11423.8	± 6.9
$A_{Rel\,1}$[%]	100.0000	± 0.0000
$I_{Rel\,1}$[%]	100.0000	± 0.0000
$Bkgr_{Dec}$[kCnts]	0.0027	± 0.0003
$\tau_{Av\,Int}$[ns]	4955.3	± 2.1
$\tau_{Av\,Amp}$[ns]	4955.3	± 2.1

Figure S34. Left: Time-resolved photoluminescence decay of [Pt(naphen)Cl] in an Ar-purged CH_2Cl_2 solution at 298 K (c = 10^{-5} M), including the residuals (λ_{ex} = 376 nm, λ_{em} = 640 nm). Right: Fitting parameters including pre-exponential factors and confidence limits.

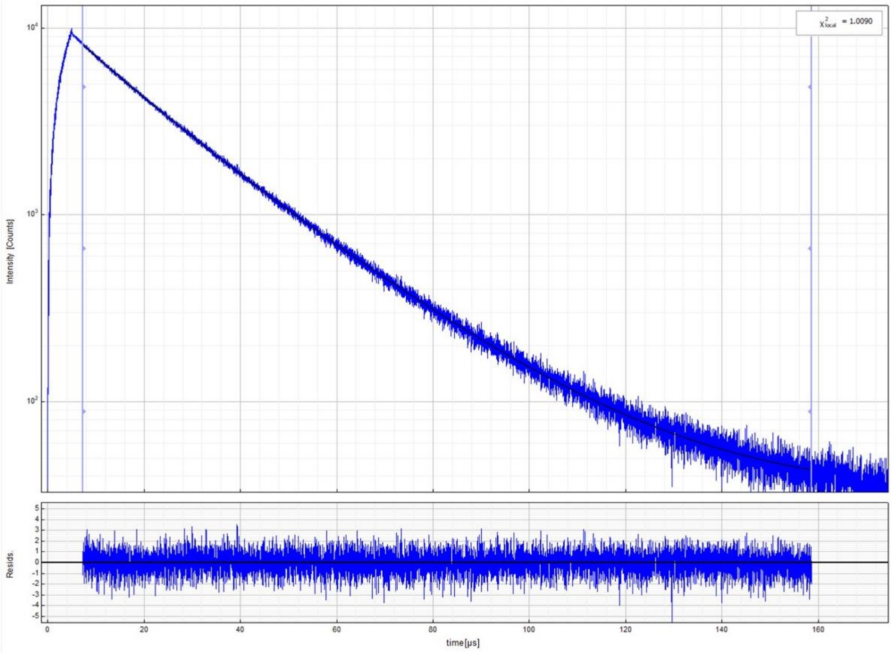

Parameter	Value	Error
A_1[kCnts/Chnl]	5.05	± 0.47
τ_1[ns]	24633	± 590
I_1[kCnts]	7777	± 541
$A_{Rel\,1}$[%]	62.6	± 5.7
$I_{Rel\,1}$[%]	75.0	± 5.4
A_2[kCnts/Chnl]	3.01	± 0.46
τ_2[ns]	13763	± 759
I_2[kCnts]	2591	± 558
$A_{Rel\,2}$[%]	37.4	± 5.7
$I_{Rel\,2}$[%]	25.0	± 5.4
$Bkgr_{Dec}$[kCnts]	0.0323	± 0.0012
$\tau_{Av\,Int}$[ns]	21916	± 68
$\tau_{Av\,Amp}$[ns]	20572	± 50

Figure S35. Left: Time-resolved photoluminescence decay of [Pt(naphen)Cl] in a frozen CH_2Cl_2:MeOH 1:1 glassy matrix at 77 (c = 10^{-5} M), including the residuals (λ_{ex} = 376 nm, λ_{em} = 620 nm). Right: Fitting parameters including pre-exponential factors and confidence limits.

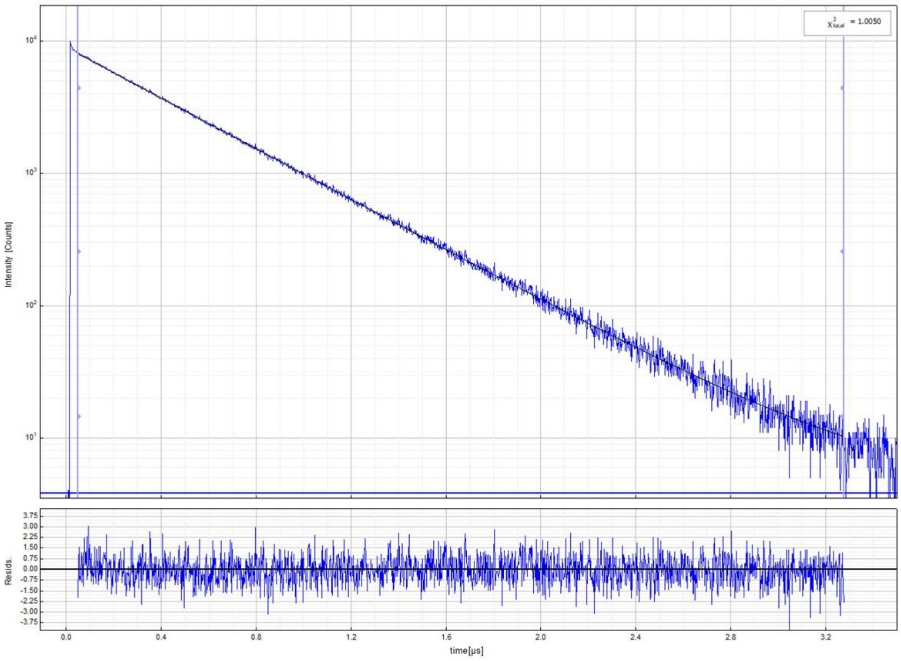

Parameter	Value	Error
A_1[kCnts/Chnl]	8.046	± 0.011
τ_1[ns]	452.56	± 0.91
I_1[kCnts]	1820.6	± 2.7
$A_{Rel\,1}$[%]	100.0000	± 0.0000
$I_{Rel\,1}$[%]	100.0000	± 0.0000
$Bkgr_{Dec}$[kCnts]	0.0038	± 0.0005
$\tau_{Av\,Int}$[ns]	452.56	± 0.91
$\tau_{Av\,Amp}$[ns]	452.56	± 0.91

Figure S36. Left: Time-resolved photoluminescence decay of [Pt(naphen)C≡CPh] in an air-equilibrated CH_2Cl_2 solution at 298 K (c = 10^{-5} M), including the residuals (λ_{ex} = 376 nm, λ_{em} = 650 nm). Right: Fitting parameters including pre-exponential factors and confidence limits.

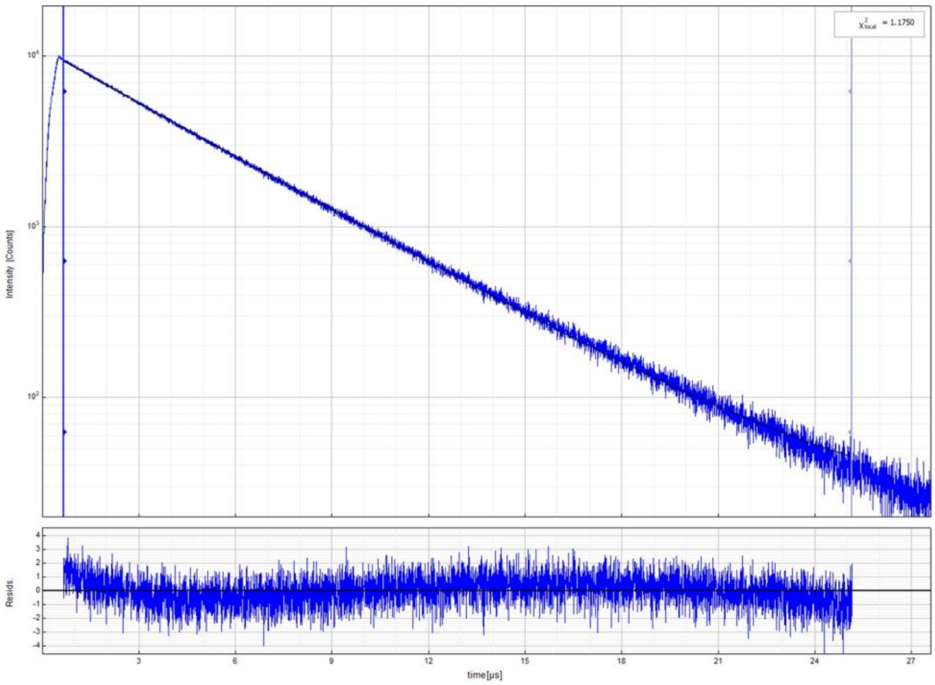

Parameter	Value	Error
A_1[kCnts/Chnl]	9.317	± 0.012
τ_1[ns]	4146.4	± 3.1
I_1[kCnts]	9657.7	± 8.4
$A_{Rel\,1}$[%]	100.0000	± 0.0000
$I_{Rel\,1}$[%]	100.0000	± 0.0000
$Bkgr_{Dec}$[kCnts]	0.0195	± 0.0005
$\tau_{Av\,Int}$[ns]	4146.4	± 3.1
$\tau_{Av\,Amp}$[ns]	4146.4	± 3.1

Figure S37. Left: Time-resolved photoluminescence decay of [Pt(naphen)C≡CPh] in an Ar-purged CH_2Cl_2 solution at 298 K (c = 10^{-5} M), including the residuals (λ_{ex} = 376 nm, λ_{em} = 650 nm). Right: Fitting parameters including pre-exponential factors and confidence limits.

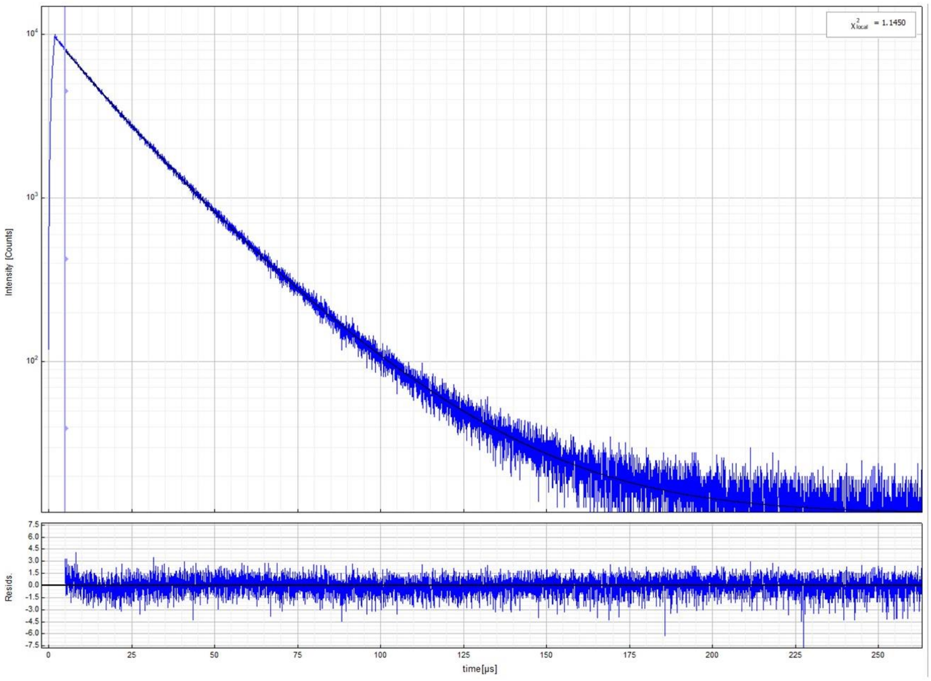

Parameter	Value	Error
A_1[kCnts/Chnl]	1.93	± 0.15
τ_1[ns]	29775	± 503
I_1[kCnts]	1799	± 103
$A_{Rel\,1}$[%]	24.3	± 1.8
$I_{Rel\,1}$[%]	36.9	± 2.1
A_2[kCnts/Chnl]	6.02	± 0.14
τ_2[ns]	16378	± 188
I_2[kCnts]	3080	± 102
$A_{Rel\,2}$[%]	75.7	± 1.8
$I_{Rel\,2}$[%]	63.1	± 2.1
$Bkgr_{Dec}$[kCnts]	0.0118	± 0.0001
$\tau_{Av\,Int}$[ns]	21318	± 30
$\tau_{Av\,Amp}$[ns]	19636	± 28

Figure S38. Left: Time-resolved photoluminescence decay of [Pt(naphen)C≡CPh] in a frozen CH_2Cl_2:MeOH 1:1 glassy matrix at 77 (c = 10^{-5} M), including the residuals (λ_{ex} = 376 nm, λ_{em} = 610 nm). Right: Fitting parameters including pre-exponential factors and confidence limits.

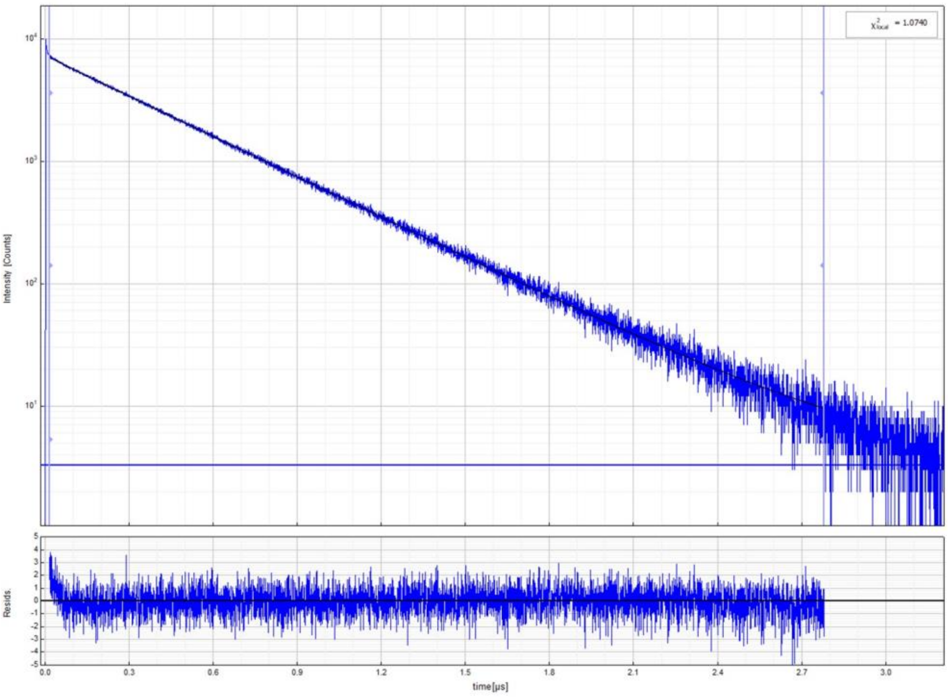

Parameter	Value	Error
A_1[kCnts/Chnl]	7.012	± 0.012
τ_1[ns]	394.03	± 0.60
I_1[kCnts]	5396.1	± 4.3
$A_{Rel\,1}$[%]	100.0000	± 0.0000
$I_{Rel\,1}$[%]	100.0000	± 0.0000
$Bkgr_{Dec}$[kCnts]	0.0033	± 0.0003
$\tau_{Av\,Int}$[ns]	394.03	± 0.60
$\tau_{Av\,Amp}$[ns]	394.03	± 0.60

Figure S39. Left: Time-resolved photoluminescence decay of [Pt(thnaphen)Cl] in an air-equilibrated CH_2Cl_2 solution at 298 K (c = 10^{-5} M), including the residuals (λ_{ex} = 376 nm, λ_{em} = 590 nm). Right: Fitting parameters including pre-exponential factors and confidence limits.

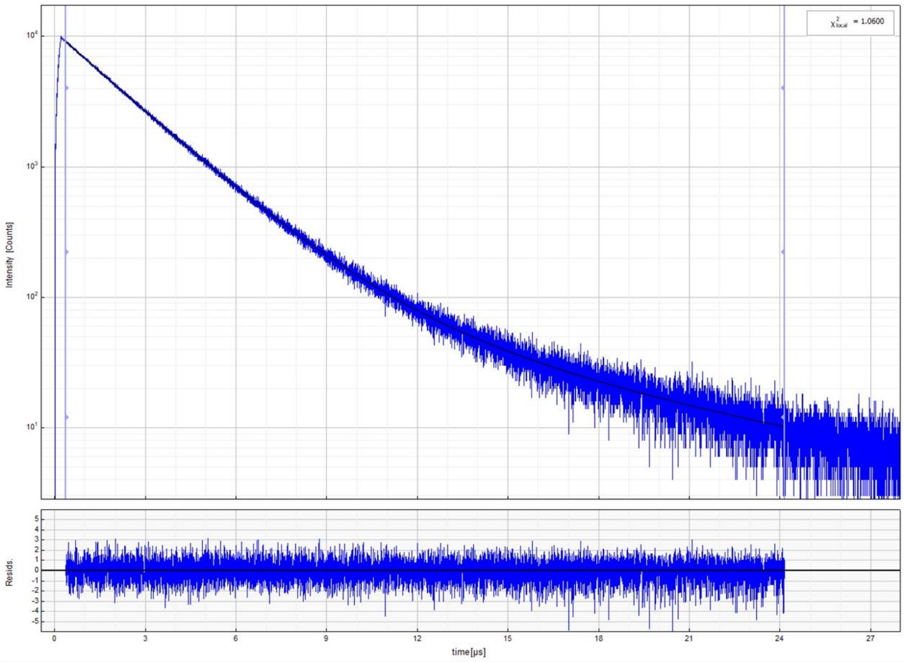

Parameter	Value	Error
A_1[kCnts/Chnl]	0.236	± 0.037
τ_1[ns]	6877	± 572
I_1[kCnts]	812	± 48
$A_{Rel\,1}$[%]	2.59	± 0.41
$I_{Rel\,1}$[%]	8.09	± 0.50
A_2[kCnts/Chnl]	8.863	± 0.035
τ_2[ns]	2080.6	± 8.1
I_2[kCnts]	9220	± 73
$A_{Rel\,2}$[%]	97.41	± 0.41
$I_{Rel\,2}$[%]	91.91	± 0.50
$Bkgr_{Dec}$[kCnts]	0.0027	± 0.0015
$\tau_{Av\,Int}$[ns]	2469	± 34
$\tau_{Av\,Amp}$[ns]	2205.0	± 6.0

Figure S40. Left: Time-resolved photoluminescence decay of [Pt(naphen)C≡CPh] in an Ar-purged CH_2Cl_2 solution at 298 K (c = 10^{-5} M), including the residuals (λ_{ex} = 376 nm, λ_{em} = 590 nm). Right: Fitting parameters including pre-exponential factors and confidence limits.

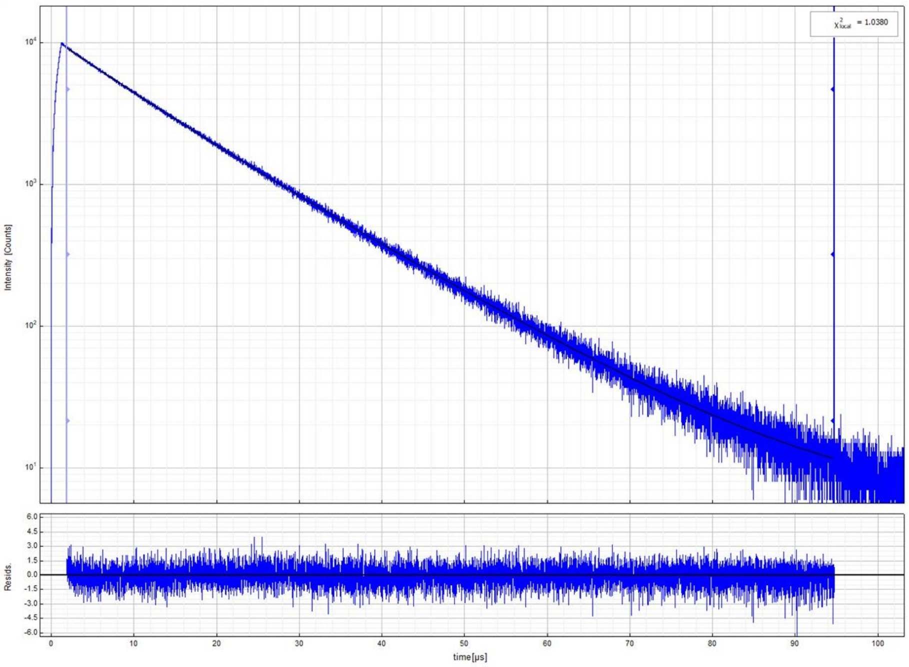

Parameter	Value	Error
A_1[kCnts/Chnl]	2.23	± 0.24
τ_1[ns]	16250	± 334
I_1[kCnts]	4540	± 395
$A_{Rel\,1}$[%]	24.5	± 2.6
$I_{Rel\,1}$[%]	34.2	± 3.0
A_2[kCnts/Chnl]	6.88	± 0.23
τ_2[ns]	10138	± 119
I_2[kCnts]	8720	± 387
$A_{Rel\,2}$[%]	75.5	± 2.6
$I_{Rel\,2}$[%]	65.8	± 3.0
$Bkgr_{Dec}$[kCnts]	0.0026	± 0.0001
$\tau_{Av\,Int}$[ns]	12231	± 13
$\tau_{Av\,Amp}$[ns]	11637	± 14

Figure S41. Left: Time-resolved photoluminescence decay of [Pt(thnaphen)Cl] in a frozen CH_2Cl_2:MeOH 1:1 glassy matrix at 77 (c = 10^{-5} M), including the residuals (λ_{ex} = 376 nm, λ_{em} = 540 nm). Right: Fitting parameters including pre-exponential factors and confidence limits.

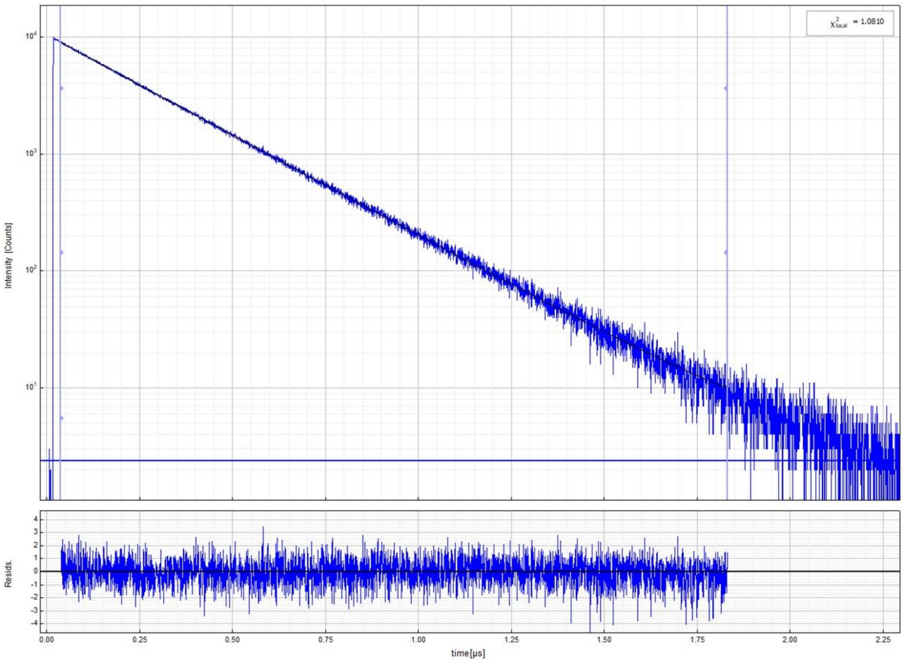

Parameter	Value	Error
A_1[kCnts/Chnl]	8.977	± 0.012
τ_1[ns]	253.23	± 0.20
I_1[kCnts]	4546.6	± 4.4
$A_{Rel\,1}$[%]	100.0000	± 0.0000
$I_{Rel\,1}$[%]	100.0000	± 0.0000
$Bkgr_{Dec}$[kCnts]	0.0024	± 0.0002
$\tau_{Av\,Int}$[ns]	253.23	± 0.20
$\tau_{Av\,Amp}$[ns]	253.23	± 0.20

Figure S42. Left: Time-resolved photoluminescence decay of [Pt(thnaphen)C≡CPh] in an air-equilibrated CH_2Cl_2 solution at 298 K (c = 10^{-5} M), including the residuals (λ_{ex} = 376 nm, λ_{em} = 600 nm). Right: Fitting parameters including pre-exponential factors and confidence limits.

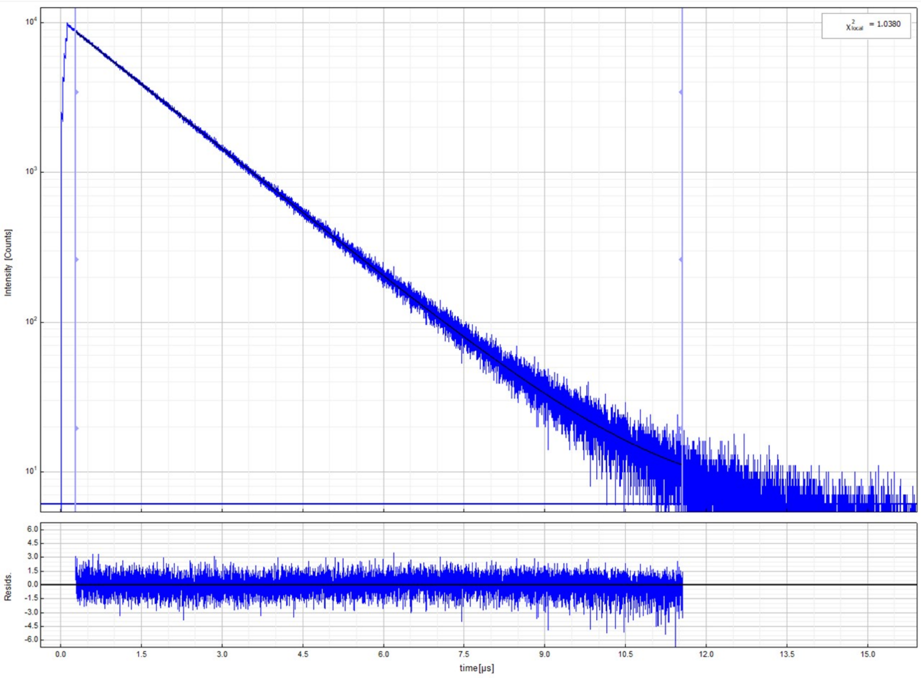

Parameter	Value	Error
A_1[kCnts/Chnl]	8.7309	± 0.0068
τ_1[ns]	1512.02	± 0.99
I_1[kCnts]	13201.4	± 7.7
$A_{Rel\,1}$[%]	100.0000	± 0.0000
$I_{Rel\,1}$[%]	100.0000	± 0.0000
$Bkgr_{Dec}$[kCnts]	0.0061	± 0.0002
$\tau_{Av\,Int}$[ns]	1512.02	± 0.99
$\tau_{Av\,Amp}$[ns]	1512.02	± 0.99

Figure S43. Left: Time-resolved photoluminescence decay of [Pt(naphen)C≡CPh] in an Ar-purged CH_2Cl_2 solution at 298 K (c = 10^{-5} M), including the residuals (λ_{ex} = 376 nm, λ_{em} = 600 nm). Right: Fitting parameters including pre-exponential factors and confidence limits.

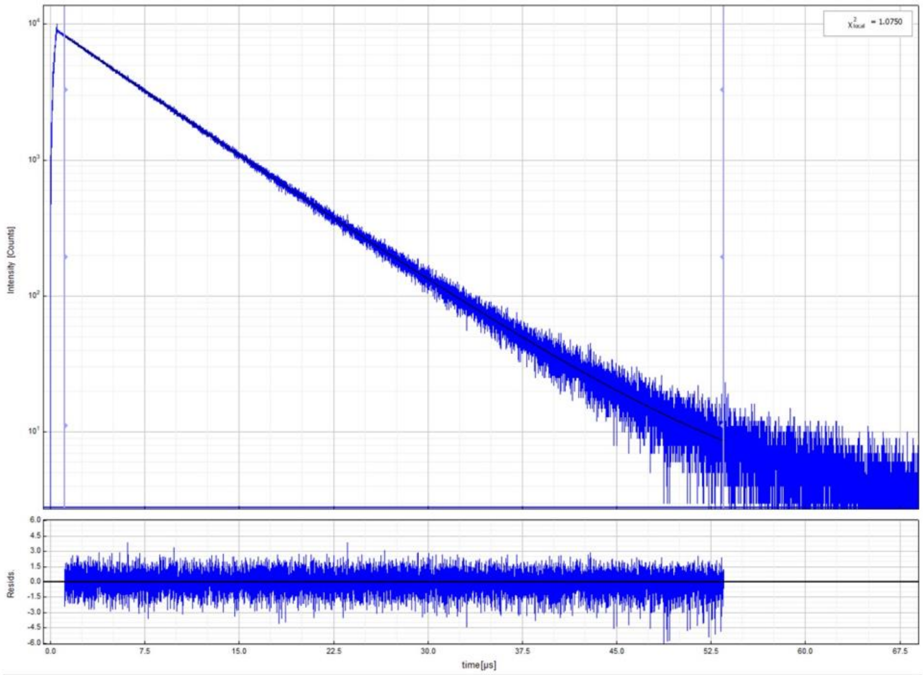

Parameter	Value	Error
A_1[kCnts/Chnl]	1.096	± 0.018
τ_1[ns]	9141	± 40
I_1[kCnts]	2505	± 33
$A_{Rel\,1}$[%]	13.36	± 0.22
$I_{Rel\,1}$[%]	17.81	± 0.24
A_2[kCnts/Chnl]	7.109	± 0.019
τ_2[ns]	6505.1	± 5.8
I_2[kCnts]	11562	± 36
$A_{Rel\,2}$[%]	86.64	± 0.22
$I_{Rel\,2}$[%]	82.19	± 0.24
$Bkgr_{Dec}$[kCnts]	0.0028	± 0.0002
$\tau_{Av\,Int}$[ns]	6974.6	± 3.2
$\tau_{Av\,Amp}$[ns]	6857.3	± 2.2

Figure S44. Left: Time-resolved photoluminescence decay of [Pt(thnaphen)C≡Ph] in a frozen CH_2Cl_2:MeOH 1:1 glassy matrix at 77 (c = 10^{-5} M), including the residuals (λ_{ex} = 376 nm, λ_{em} = 550 nm). Right: Fitting parameters including pre-exponential factors and confidence limits.

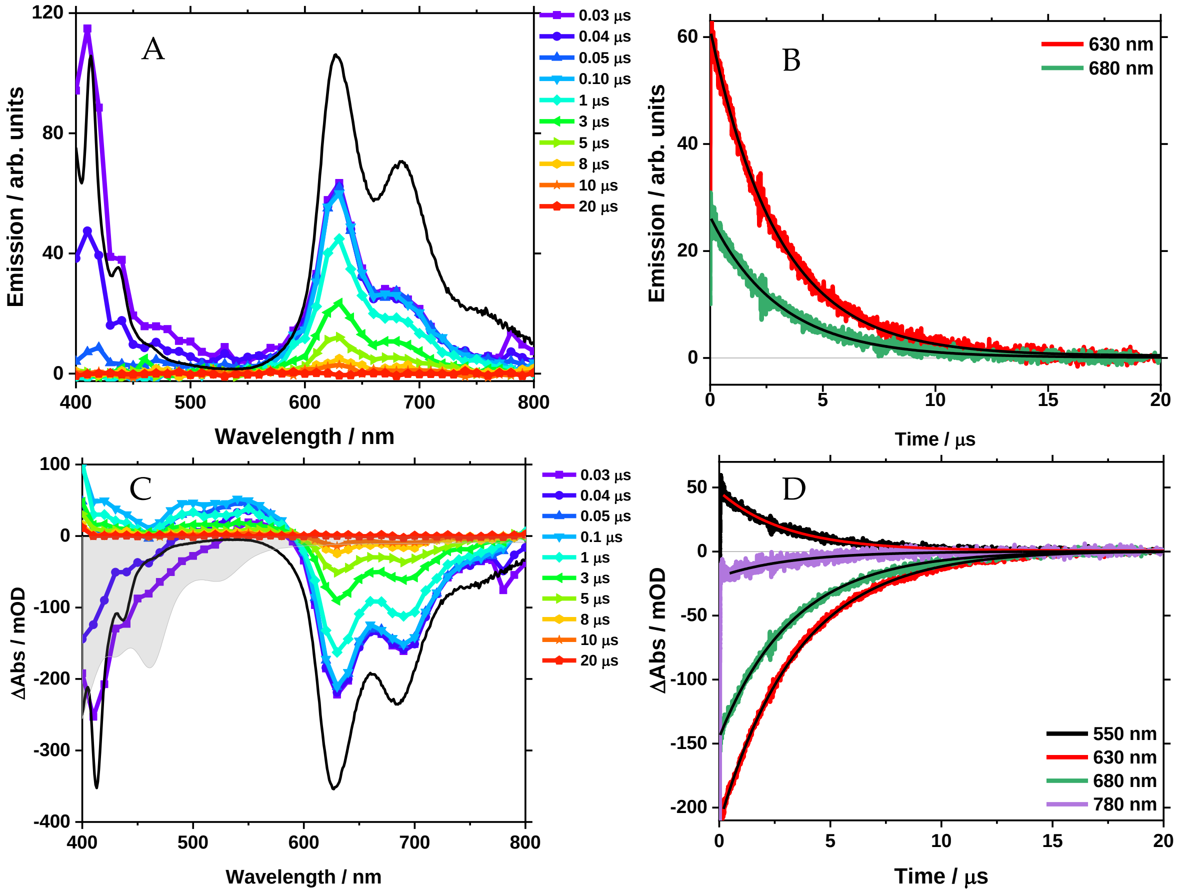

Figure S45. Left: Nanosecond time-resolved emission spectra superimposed to the inverted steady-state spectrum in black (A) and transient absorption spectra (B) of [Pt(naphen)Cl] in CH_2Cl_2 at selected time points. Black/grey line refers to superimposed inverted steady state emission/absorption spectra respectively. Right: Decay fits at selected wavelengths. Please note: The figure presents data, which were recorded in the presence of trace amount of ligand. The residual free ligand in the sample leads to the short-lived emission band centered at 410 nm.

Supporting Tables

Table S1. UV-vis absorptions of the ligands and [Pt(L)X] complexes [a].

[Pt(L)X] L =	X	λ_1 (ε)	λ_2 (ε)	λ_3 (ε)	λ_4 (ε)	λ_5 (ε)	λ_6 (ε)	λ_7 (ε)	λ_8 (ε)	λ_9 (ε)	λ_{10} (ε)	cut-off (nm)
Hnaphen		242 (1.25)	256 (1.17)	289sh (2.09)	297 (2.48)	308 (1.80)	326 (0.49)	336sh (0.42)	351 (0.29)	369 (0.26)	388 (0.27)	
Pt naphen	Cl	255 (21.8)	284 (18.2)	300 (24.7)	323 (9.1)	354 (6.0)	380 (5.3)	401 (3.4)	428 (2.6)	461 (2.7)	523 (0.9)	600
Pt naphen	C≡CPh	256 (28.7)	284 (24.0)	301 (26.8)	321 (9.8)	361 (5.1)	378 (11.2)	390 (5.1)	433 (2.5)	464 (3.3)	534 (1.2)	606
Hthnaphen		241sh (2.09)	247 (2.23)	285 (0.89)	295 (0.79)	330 (1.43)	338 (1.45)	352sh (0.37)				
Pt thnaphen	Cl	246 (17.7)	266 (16.9)	291 (20.0)	307 (16.1)	338 (11.9)	361 (10.4)	383 (9.8)	428 (2.3)	452 (1.0)	524 (0.1)	563
Pt thnaphen	C≡CPh	252 (26.8)	286 (33.3)	304 (10.8)	346 (14.9)	357 (13.9)	381 (11.2)	387 (9.7)	430 (5.3)	451 (5.0)	533 (0.3)	563
Hbdq		252 (2.33)	286sh (2.93)	298 (5.56)	303sh (4.23)	317sh (1.18)	333 (0.73)	352 (0.36)	372 (0.45)	393 (0.67)		
Pt bdq	Cl	254 (25.0)	277 (25.5)	298 (20.4)		324 (12.2)	360 (4.6)	384 (4.1)	433 (4.8)	461 (7.7)	493 (0.6)	568
H_2dba [b]		289 (63.3)	302 (57.0)	338 (8.6)	324 (8.3)	355 (6.5)	372 (9.0)	394 (13.5)				
Pt dba [b]	dmso	-	296 (56.5)	-	327 (9.1)	341 (8.1)	386 (3.7)	409 (3.9)	461 (1.0)	498 (1.8)	529 (2.3)	580

[a] Absorption maxima λ_{abs} in nm (ε in 10^3 M^{-1} cm^{-1}) in CH_2Cl_2 (298 K). [b] From Ref. [1], measured in THF.

Reference

(1) S. Garbe, M. Krause, A. Klimpel, I. Neundorf, P. Lippmann, I. Ott, D. Brünkink, C. A. Strassert, N. L. Doltsinis, A. Klein, Cyclometalated Pt Complexes of CNC Pincer Ligands: Luminescence and Cytotoxic Evaluation. *Organometallics* **2020**, *39*, 746–756.
